# A bioinformatic study of antimicrobial peptides identified in the Black Soldier Fly (BSF) *Hermetia illucens* (Diptera: Stratiomyidae)

**DOI:** 10.1038/s41598-020-74017-9

**Published:** 2020-10-09

**Authors:** Antonio Moretta, Rosanna Salvia, Carmen Scieuzo, Angela Di Somma, Heiko Vogel, Pietro Pucci, Alessandro Sgambato, Michael Wolff, Patrizia Falabella

**Affiliations:** 1 Department of Sciences, University of Basilicata, Via dell’Ateneo Lucano 10, 85100 Potenza, Italy; 2 Department of Chemical Sciences, University Federico II of Napoli, Via Cinthia 6, 80126 Napoli, Italy; 3 Department of Entomology, Max Planck Institute for Chemical Ecology, Hans-Knöll-Straße 8, 07745 Jena, Germany; 4 CEINGE Advanced Biotechnology, Via Gaetano Salvatore 486, Naples, Italy; 5 Centro di Riferimento Oncologico della Basilicata (IRCCS-CROB), Rionero in Vulture, PZ Italy; 6 Department of Translational Medicine and Surgery, Università Cattolica del Sacro Cuore, Rome, Italy; 7 Institute of Bioprocess Engineering and Pharmaceutical Technology, Technische Hochschule Mittelhessen, Wiesenstrasse 14, 35390 Giessen, Germany

**Keywords:** Entomology, Computational biology and bioinformatics

## Abstract

Antimicrobial peptides (AMPs) play a key role in the innate immunity, the first line of defense against bacteria, fungi, and viruses. AMPs are small molecules, ranging from 10 to 100 amino acid residues produced by all living organisms. Because of their wide biodiversity, insects are among the richest and most innovative sources for AMPs. In particular, the insect *Hermetia illucens* (Diptera: Stratiomyidae) shows an extraordinary ability to live in hostile environments, as it feeds on decaying substrates, which are rich in microbial colonies, and is one of the most promising sources for AMPs. The larvae and the combined adult male and female *H. illucens* transcriptomes were examined, and all the sequences, putatively encoding AMPs, were analysed with different machine learning-algorithms, such as the Support Vector Machine, the Discriminant Analysis, the Artificial Neural Network, and the Random Forest available on the CAMP database, in order to predict their antimicrobial activity. Moreover, the iACP tool, the AVPpred, and the Antifp servers were used to predict the anticancer, the antiviral, and the antifungal activities, respectively. The related physicochemical properties were evaluated with the Antimicrobial Peptide Database Calculator and Predictor. These analyses allowed to identify 57 putatively active peptides suitable for subsequent experimental validation studies.

## Introduction

With over one million described species, insects represent the most diverse as well as the largest class of organisms in the world, due to their ability to adapt to recurrent changes and to their resistance against a wide spectrum of pathogens^1^. Their immune system, exclusively based on the innate, well-developed immune response, allows a general and rapid response to various invading organisms^2, 3^. The humoral immune response includes the enzymatic cascade that regulates the activation of coagulation and melanization of the hemolymph, the production of reactive oxygen (ROS) and nitrogen (RNS) species, and the production of antimicrobial peptides (AMPs)^4^.

Today, the problem of antibiotic resistance represents one of the greatest threats in the medical field^4^. The constant need to find alternative solutions has increased the interest in AMPs over time. AMPs are small molecules, consisting of 10–100 amino acids, that have been identified in many organisms such as bacteria, fungi, plants, vertebrates and invertebrates, including insects^5^. They are cationic molecules that exhibit activities against bacteria, fungi, viruses, and parasites^5^. In addition to these known activities, many peptides also exert a cytotoxic effect against cancer cells^6^.

The discovery of the first AMP derived from insects, dates back to 1980s, when Boman et al.^7^ identified and isolated the first cecropin from the lepidopteran *Hyalophora cecropia*. Since then, many other AMPs have been discovered. Due to their high biodiversity, insects are considered to be among the richest and most innovative sources for these molecules. Insect AMPs can be classified into four families: α-helical peptides (e.g. cecropins), cysteine-rich peptides (e.g. defensins), proline-rich peptides, and glycine-rich peptides^8^. Despite their diversity, AMPs share two common features: the tendency to adopt an amphipathic conformation and the presence of a large number of basic residues, which determine the net positive charge at a neutral pH^9^. The established electrostatic forces between the positive amino acid residues of a peptide and the negative charges exposed on microorganism cell surfaces allow their interaction with bacterial membranes. Moreover, the cationic nature of these peptides allows the interaction with the negatively charged molecules exposed on cancer cell surfaces, such as phospholipid phosphatidylserine (PS), O-glycosylated mucins, sialylated gangliosides, and heparin sulfate, in contrast to the typical zwitterionic nature of the normal mammalian membranes^6,10,11^. According to their mechanism of action, AMPs can be grouped in two categories^12^, (1) the membranolytic mechanism, described by three different putative models: “carpet”, “toroidal” and “barrel-stave” model^13^, and (2) the non-membranolytic one, characterised by their direct interaction with intracellular targets such as DNA, RNA and proteins^14–16^.

To date, more than 3000 AMPs have been discovered and reported to the Antimicrobial Peptide Database (APD, https://aps.unmc.edu/AP/), which contains exactly 3104 AMPs from six kingdoms: 343 from bacteria, 5 from archaea, 8 from protists, 20 from fungi, 349 from plants, and 2301 from animals. The amount of AMPs in insects varies according to the species, i.e. more than 50 AMPs have been found in the invasive ladybird *Harmonia axyridis*^17^, whereas none was identified in the pea aphid *Acyrthosiphon pisum*^18^. The non-pest insect *Hermetia illucens* (Diptera: Stratiomyidae), also known as the Black Soldier Fly (BSF), is among the most promising sources for AMPs being able to live in hostile environments rich in microbial colonies^19^. In this study, we have analysed the larvae and the combined adult male and female *H. illucens* transcriptomes in order to identify AMPs, which were then analysed with the CAMP (Collection of Antimicrobial Peptides) database (https://www.camp.bicnirrh.res.in/)^20–23^. Moreover, the iACP online tool (https://lin.uestc.edu.cn/server/iACP) was used to predict the anticancer activity of the identified peptides while the AVPpred (https://crdd.osdd.net/servers/avppred) server was used to predict the antiviral activity of the identified peptides while the Antifp server (https://webs.iiitd.edu.in/raghava/antifp) was used to predict their antifungal activity, and their physicochemical properties were evaluated with the Antimicrobial Peptide Database Calculator and Predictor (APD3).

## Results

### De novo* transcriptome assembly and gene identification*

A Next-Generation sequencing (RNAseq) of the RNA isolated from larvae and combined adult male and female *H. illucens* transcriptomes was performed for an unambiguous identification of the peptide candidates. Sequencing and de novo assembly of the transcriptomes led to the identification of 25,197 unique nucleotide sequences (contigs) in the larvae transcriptome, and 78,763 contigs in the combined adults. These contigs were functionally annotated using Blast2GO software (https://www.blast2go.org). A total of 68 genes, encoding putative AMPs in the *H. illucens* transcriptomes, were finally identified.

### Antimicrobial, anticancer, antiviral and antifungal activity prediction

All identified 68 sequences, encoding putative AMPs, were analysed in silico by the four machine-learning algorithms, such as Support Vector Machine (SVM), Discriminant Analysis (DA), Artificial Neural Network (ANN), and Random Forest (RF), available on the free online CAMP database, in order to predict their antimicrobial activity. The results are shown in Table 1. Table 2 reports the anticancer and non-anticancer scores obtained using the iACP tool. Table 3 shows the results obtained with the AVPpred server to predict the antiviral activity and with the Antifp server used to predict the antifungal activity. These analyses allowed the identification of 57 putatively active peptides: 13 sequences were predicted to be only antimicrobial while the others showed different combinations of antimicrobial, antiviral, anticancer or antifungal activity. In particular, 22 were both putative antimicrobial and anticancer; eight were both putative antimicrobial and antiviral; two were both putative antimicrobial and antifungal; seven were putative antimicrobial, anticancer and antiviral; one was putative antimicrobial antifungal and antiviral; two were putative antimicrobial, anticancer and antifungal while two potentially cover the complete range of analyzed biological activities (antimicrobial, anticancer, antifungal and antiviral). The remaining 11 did not show any activity according to the in silico investigation. In Supplementary Table S1 all the predicted activities are listed.

**Table 1:** Prediction of the antimicrobial activity through the CAMP database.

Peptide	Sequence	SVM	RF	ANN	DA
Hill_BB_C14202	KRFTKCTLARELFQRGIPKSELPDWVCLVRWESNYQTNAMNKNNRDGSWDYGLFQINDKWWCKGHIKSHNACGLSCNELLKDDISKAVTCARLIKRQQGFRAWYGWLNHCTKVKPSIHECF	1.000	0.800	AMP	1.000
Hill_BB_C3566	AKMSRCGVANMLLKYGFPRKDLADWVCLIEHESSFRTNVVGPPNTDGSRDYGLFQINSRYWCSGDGPSHNMCRIPCRMLLSNDMTHSIRCAVTVFRKQGLSAWYGWSGHCQGNAPSVENCFRSYNNLYYGK	1.000	0.916	AMP	1.000
Hill_BB_C1152	RYGFPRNQLADWICLVEWESSFRTDAVGPPNGDGSRDWGLFQINDRYWCQSANYGNSHNICGVSCERLLSDDITTAVNCVRKIYAAHGFSGWNAWTQHCHSPSSVEHCFVESDCLPGGVSFDKHWL	1.000	0.8045	AMP	1.000
Hill_BB_C1153	ASGRQFERCELARILHNRYGFPRNQLADWICLVEWESSFRTNAVGPPNSDGSRDWGLFQINDRYWCKSSNYRNSHNMCGVSCEHLLSDDITTAVNCVRKIYAAHGFSGWNAWTQH	1.000	0.918	AMP	1.000
Hill_BB_C2676	TVYSRCGFAQTLYYDYGVTDMNTLANWVCLVQYESSFNDQAVGAINYNGTQDFGLFQINNKYWCQGAVSSSDSCGIACTSLLGNLSASWSCAQLVYQQQGFSAWYGWLNNCNGTAPSVADCF	1.000	0.611	AMP	1.000
Hill_BB_C269	KVFTRCQLAKELIRYDFPRTFLSNWVCLIESESGRSTSKTLQLPNTSANYGIFQINSKTWCRKGRKGGLCEMKCEDFLNDDISDDARCAKQIYNRHGFQGWPGWVNKCRGRALPDVLKC	1.000	0.8725	AMP	1.000
Hill_BB_C1169	SNGPRDYGLFQINNQYWCQGNVKSANECHIACTSLLSDDITHALNCAKKIKAQQGFKAWYGWLNYCQKSKPSVKECF	0.937	0.8045	AMP	0.993
Hill_BB_C779	KVYTRCEMARILYHDHGVKNLTTLANWVCLIEHESGFNDEAVGALNSNGTRDYGLFQINNKYWCKGNVASSDSCKIACTALLGNVDASWKCAQLVYKEQGFKAWYGW	1.000	0.7555	AMP	1.000
Hill_LB_C36111	KQFNKCSLATELSRLGVPKSELPDWVCLVQHESNFKTNWINKKNSNGSWDFGLFQINDKWWCEGHIRSHNTCNVKCEELVTEDIEKALECAKVIKRERGYKAWYGWLNNCQNKKPSVDECF	1.000	0.8235	AMP	1.000
Hill_LB_C12085	KTFTKCSLAKTLYAHGIPKSELPDWVCLVQHESGFRTDAVGALNSNGTRDYGLFQINNKYWCKGNISSYNECNIACSALLSDDI	0.890	0.871	AMP	0.987
Hill_BB_C1290	QLNIQGGAKSPLSDFDLNVQGGARKYYNNGHKPLHGTEDYNQHLGGPYGYSRPNFGGGLLFTHRFKLCSLSKLLIVC	0.581	0.5055	AMP	0.554
Hill_BB_C7347	QLNIQGGGSPHSGFNLSIQGQKKLWESNNKRNTLHGTGQYSQHF	0.307	0.374	NAMP	0.031
Hill_BB_C9109	QIFAQGGGSPGKGYDIYAQGRAKLWESQNQRNSLHGTASYSQHLGGPYGNSRPNVGGGLIFTHRF	0.351	0.6175	AMP	0.270
Hill_BB_C11804	QLNIQGGGSPHSGFNLSIQGQKKLWESNNKRNTLHGTGQYSQHF	0.307	0.374	NAMP	0.031
Hill_BB_C309	VSCWFENENIKASACQMSCMYRKGRRGGMCVNGVCTCSPN	0.827	0.6825	AMP	0.908
Hill_BB_C1827	TTCTHLNCKLHCVLYRKRSGRCDRFNICKCI	0.898	0.8805	AMP	0.995
Hill_BB_C5878	LSCLFENQAISAIACGASCITRKGRRGGWCSNGVCRCTPN	0.971	0.941	AMP	0.994
Hill_BB_C8756	QPYQLQYEEDGPEYARELPIEEEELPSQVVEQHHQAKRATCDLLSPFKVGHAACVLDGFAMGRRGGWC	0.266	0.0085	NAMP	0.037
Hill_BB_C13793	KESSDPDSALYSDIHPRFRRQLPCDYLSGLGFGEDACNTDCIAKGHKSGFCTGLVCRCRTL	0.503	0.5453	AMP	0.645
NHill_AD_C73537	GQSEASWWKKVFKPVEKLGQRVRDATIQGIGIAQQGANVLATVRGGPPQ	0.633	0.870	AMP	0.904
NHill_AD_C16493	GQSEAGWWKRVFKPVEKFGQRVRDAGVQGIAIAQQGANVLATARGGPPQQG	0.633	0.842	AMP	0.885
NHill_AD_C12927	GWWKRVFKPVEKLGQRVRDAGIQGLEIAQQGANVLATARGGPPQQG	0.672	0.9075	AMP	0.955
NHill_AD_C12928	GWWKRVFKPVERLGQRVRDAGIQGLQIAQQGANVLATVRGGPPQQG	0.773	0.911	AMP	0.969
NHill_AD_C4669	SWFKKVFKPVEKVGQRVRDAGIQGVAIAQQGANVLATARGGPPH	0.574	0.745	AMP	0.899
Hill_BB_C3195	GWWKKVFKPVEKLGQRVRDAGIQGIAIAQQGANVLATVRGGPPQ	0.868	0.9945	AMP	0.988
Hill_SB_C698	GQSEAGWWKRVFKPVEKFGQRVRDAGIQGIEIAQQGANVLATARGGPPQQG	0.558	0.718	AMP	0.770
Hill_SB_C2730	GWWKRVFKPVEKLGQRVRDAGIQGLEIAQQGANVLATVRGGPPQQG	0.700	0.9095	AMP	0.959
Hill_SB_C1875	GQGESRSLWKKIFKPVEKLGQRVRDAGIQGIAIAQQGANVLATVRGGPPQ	0.714	0.9115	AMP	0.949
Hill_BB_C5151	GQSESRSLWKKLFKPVERAGQRIRDATIKGIVIAQQGANVLATIRGGPAIPPGQG	0.641	0.944	AMP	0.935
Hill_BB_C390	FNNLPICVEGLAGDIGSILLGVESDIGALAGAIANLALIAGECAAQGEAGAAICA	0.946	0.685	AMP	0.822
NHill_AD_C53857	CINNGDGCQPDGRQGNCCSGYCHKEPGWVTGYCR	0.811	0.742	AMP	0.973
NHill_AD_C49215	CIANGNGCQPDGRQGNCCSGFCYKQRGWVAGYCRRR	0.961	0.8735	AMP	0.999
Hill_BB_C2323	QLNIQGGGSPHSGFDLSVQGRAKIWESDNGRNTLYGTGQYGQHLGGPYGNSEPSFGGGLMFSHRF	0.163	0.048	NAMP	0.007
Hill_BB_C7345	SIDDLTLSEDGEDHVEIITDDEVQRAKR	0.456	0.1395	NAMP	0.024
Hill_BB_C7346	QLNIQGGGSPHSGFDLNVQGRAKIWESNNGRNTLHGTGEYSQHLGGPYGNSRPNFGGGLLFTHRF	0.223	0.1105	NAMP	0.019
Hill_BB_C11803	QLNIQGGGSPHSGFNLSIQGQKKLWESNNKRNTLHGTGQYSQHFGGPYGNSRPNFGGGLVFTHRF	0.278	0.3295	AMP	0.030
Hill_BB_C21232	QLNIQGGSKSTFLILISMSKVVRESNNGHETLHGTGDYNQHLGGPYGNSQPNFGGELLFTHRFKLCSLSKLLIVCVFSKCRK	0.749	0.8505	AMP	0.865
NHill_AD_C17624	QIFAQGGGSPGKGYDIYAQGRAKLWESQNQRNSLHGTASYSQHLGGPYGNSRPNVGGGLTFTHRF	0.284	0.515	NAMP	0.170
Hill_LB_C16634	IKCTASICTQICRILKYKCGYCASASRCVCLK	0.992	0.913	AMP	0.999
Hill_LB_C37730	AFAFDVTRKINPETSAVERPEVSEYPEIPKGTKLQEFVMMDIEIEEEGADNRAETIQRIKCVPSQCNQICRVLGKKCGYCKNASTCVCLG	0.988	0.9565	AMP	0.984
Hill_BB_C46948	RKCTASQCTRVCKKLGYKRGYCQSSTKCVC	0.968	0.9375	AMP	0.999
Hill_BB_C16137	MNIQGNAVSNPAGGQDVTVTAGKQFGSDNANITAGGFAGGNTLRGPPNAGVFASANANGHSLSVSKTVVPGISSTTSHGASANLFR	0.886	0.8225	AMP	0.758
Hill_BB_C16883	QLSGSITPDMAGGNNVNIMASKFLGNPNHNIGGGVFASGNTRSNTPSLGAFGTLNLKDHSLGVSKTITPGVSDTFSQNARLIILKTPDHRVDANVFNSHTRLNNGFAFDKRGGSLDYTHRAGHSLSLGASHIPKFGTTAELTGKANLWKSPSGLSTFDLTGSAS	1.000	0.9275	AMP	1.000
Hill_BB_C10074	SPQDGRRGSASVTVNNESRRGTDVRADLNARLWEGNNRRSSLDANAYYQRHFGGPMGTGRPDAGVGLNFRHRF	0.400	0.4375	NAMP	0.566
Hill_BB_C9237	MNIQGNAVSNPAGGQDVTVTAGKQFGSDNTNITAGAFAGGNTLRGPPNAGVFASANANSHSLSVSKTVVPGVSATTSHAASANLFRNDQHSVNAQAFSSATKLNDGFQFKQHGAGLNYNNANGHGASIGVNKIPGFGSSMDVGARANIFQNPNTSFDVMANSRTHLSGPFQGKTNFGVSAGITRRF	1.000	0.9505	AMP	1.000
NHill_AD_C40487	MNIQGNAVSNPAGGQDVTVTAGKQFGSDNTNITAGAFAGGNTLRGPPNAGVFASANANGHSLSVSKTVVPGVSSTTSHAASANLFRNDQHNVNAQAFSSATKLNDGFQFKQHGAGLNYNNANGHGASIGVNKIPGFGSSMDVGARANIFQNPNTSFDVMANSRTHLSGPFQGKTNF	1.000	0.9745	AMP	1.000
Hill_BB_C7758	AACDLFSALNVASSICAAHCLYLGYKGGYCDSKLVCVCR	0.985	0.819	AMP	0.988
Hill_BB_C14087	VTCDLLEPFLGPAPCMIHCIVRFRKRTGYCNSQNVCVCRG	0.712	0.6305	AMP	0.709
Hill_LB_C29142	ATCDLLSPFKVGHAACAAHCIARGKRGGWCDKRAVCNCRK	0.956	0.9455	AMP	0.999
Hill_BB_C308	VSCWFENENIKASACQMSCMYRKGRRGGMCVNGVCTCSPN	0.827	0.6825	AMP	0.908
Hill_BB_C1619	LSCLFENQAVSAIACGSSCIARKGRRGGYCRNGVCVCTDN	0.972	0.900	AMP	0.972
Hill_BB_C1826	TTCTHLNCKLHCLLQRKRSGRCDRFNICKCIS	0.878	0.9105	AMP	0.995
Hill_BB_C6571	ATCTNWNCRTQCIARGKRGGYCVERNICKCTS	0.950	0.9815	AMP	0.992
Hill_BB_C7081	ATCDLISGTKIENVACAAHCIAMGHKGGYCNSNLICICR	0.987	0.907	AMP	0.979
Hill_BB_C7985	FTCSNLGCKAQCIILGNRSGGCNRLGVCQCN	0.991	0.9175	AMP	0.999
Hill_BB_C7176	ATCDLLSPFKVGHAACALHCIALGRRGGWCDGRAVCNCRR	0.933	0.938	AMP	0.996
Hill_BB_C2519	ATCDLLSPFKVGHAACALHCIAMGRRGGWCDGRAVCNCRR	0.895	0.8835	AMP	0.987
Hill_BB_C8473	ATCDLLSPFGVGHAACAVHCIAMGRRGGWCDDRAVCNCRR	0.855	0.8145	AMP	0.977
Hill_BB_C34351	AMCDLLSGLNMGRSVCAMRCILKGHRGGWCDDQGVCNCRV	0.816	0.6875	AMP	0.971
Hill_BB_C4683	RPDNIEYLEDSQVAELVRHKRLSCLFENEAISALACGASCITRKGRRGGWCSNGVCHCTPN	0.734	0.5745	AMP	0.645
Hill_BB_C4977	LSCWFENEDIKATACAMSCIYRKGRKGGRCENGICRCTPN	0.828	0.7115	AMP	0.913
Hill_BB_C13326	LSCLFENQAVSAIACGASCITRKGKRGGWCSNGVCRCTPN	0.975	0.9475	AMP	0.991
Hill_BB_C7171	TTCDLISGTKIENIACAAHCIAMGHKGGYCNSNLICICR	0.981	0.8805	AMP	0.984
Hill_BB_C10649	QFDNLEDTGVEEKVRHKRLTCLFDNRPISAFACGSNCVSRKGKRGGWCVNGVCRCT	0.860	0.595	AMP	0.983
Hill_BB_C13792	KQSSDPESALYSDIHPRFRRQLPCDYLSGLGFGEDACNTDCIAKGHKSGFCTGLVCRCRTL	0.995	0.9725	AMP	0.965
Hill_BB_C15867	VTCDLLKPFFGRAPCMMHCILRFKKRTGFCSRQNVCVCR	0.826	0.5095	AMP	0.885
NHill_AD_C69719	DVSIGSCVWGGSNYVSDCNGECKRRGYKGGHCGSFLNNICWCET	0.984	0.913	AMP	0.993
Hill_BB_C49430	APQFGGQIGGFGGGGFGGGGFGPGGGFRPGGVAEFQESSSSVNVERETFDQGGFEISDSSVTSSSVSESFRD	0.012	0.2715	NAMP	0.031

From left to right are shown in order: peptide contig, peptide sequence, Support Vector Machine (SVM) score, Random Forest (RF) score, Artificial Neural Network (ANN) result and the Discriminant Analysis (DA) score.

**Table 2:** Prediction of the anticancer activity through the iACP tool.

Peptide	Sequence	Anticancer score	Non-anticancer score
Hill_BB_C14202	KRFTKCTLARELFQRGIPKSELPDWVCLVRWESNYQTNAMNKNNRDGSWDYGLFQINDKWWCKGHIKSHNACGLSCNELLKDDISKAVTCARLIKRQQGFRAWYGWLNHCTKVKPSIHECF	0.452542	0.547458
Hill_BB_C3566	AKMSRCGVANMLLKYGFPRKDLADWVCLIEHESSFRTNVVGPPNTDGSRDYGLFQINSRYWCSGDGPSHNMCRIPCRMLLSNDMTHSIRCAVTVFRKQGLSAWYGWSGHCQGNAPSVENCFRSYNNLYYGK	0.603649	0.396351
Hill_BB_C1152	RYGFPRNQLADWICLVEWESSFRTDAVGPPNGDGSRDWGLFQINDRYWCQSANYGNSHNICGVSCERLLSDDITTAVNCVRKIYAAHGFSGWNAWTQHCHSPSSVEHCFVESDCLPGGVSFDKHWL	0.744031	0.255969
Hill_BB_C1153	ASGRQFERCELARILHNRYGFPRNQLADWICLVEWESSFRTNAVGPPNSDGSRDWGLFQINDRYWCKSSNYRNSHNMCGVSCEHLLSDDITTAVNCVRKIYAAHGFSGWNAWTQH	0.322215	0.677785
Hill_BB_C2676	TVYSRCGFAQTLYYDYGVTDMNTLANWVCLVQYESSFNDQAVGAINYNGTQDFGLFQINNKYWCQGAVSSSDSCGIACTSLLGNLSASWSCAQLVYQQQGFSAWYGWLNNCNGTAPSVADCF	0.508041	0.491959
Hill_BB_C269	KVFTRCQLAKELIRYDFPRTFLSNWVCLIESESGRSTSKTLQLPNTSANYGIFQINSKTWCRKGRKGGLCEMKCEDFLNDDISDDARCAKQIYNRHGFQGWPGWVNKCRGRALPDVLKC	0.353721	0.646279
Hill_BB_C1169	SNGPRDYGLFQINNQYWCQGNVKSANECHIACTSLLSDDITHALNCAKKIKAQQGFKAWYGWLNYCQKSKPSVKECF	0.995537	0.004463
Hill_BB_C779	KVYTRCEMARILYHDHGVKNLTTLANWVCLIEHESGFNDEAVGALNSNGTRDYGLFQINNKYWCKGNVASSDSCKIACTALLGNVDASWKCAQLVYKEQGFKAWYGW	0.717440	0.282560
Hill_LB_C36111	KQFNKCSLATELSRLGVPKSELPDWVCLVQHESNFKTNWINKKNSNGSWDFGLFQINDKWWCEGHIRSHNTCNVKCEELVTEDIEKALECAKVIKRERGYKAWYGWLNNCQNKKPSVDECF	0.644890	0.355110
Hill_LB_C12085	KTFTKCSLAKTLYAHGIPKSELPDWVCLVQHESGFRTDAVGALNSNGTRDYGLFQINNKYWCKGNISSYNECNIACSALLSDDI	0.500000	0.500000
Hill_BB_C1290	QLNIQGGAKSPLSDFDLNVQGGARKYYNNGHKPLHGTEDYNQHLGGPYGYSRPNFGGGLLFTHRFKLCSLSKLLIVC	0.878792	0.121208
Hill_BB_C7347	QLNIQGGGSPHSGFNLSIQGQKKLWESNNKRNTLHGTGQYSQHF	0.005102	0.994898
Hill_BB_C9109	QIFAQGGGSPGKGYDIYAQGRAKLWESQNQRNSLHGTASYSQHLGGPYGNSRPNVGGGLIFTHRF	0.115082	0.884918
Hill_BB_C11804	QLNIQGGGSPHSGFNLSIQGQKKLWESNNKRNTLHGTGQYSQHF	0.005102	0.994898
Hill_BB_C309	VSCWFENENIKASACQMSCMYRKGRRGGMCVNGVCTCSPN	0.444002	0.555998
Hill_BB_C1827	TTCTHLNCKLHCVLYRKRSGRCDRFNICKCI	0.215222	0.784778
Hill_BB_C5878	LSCLFENQAISAIACGASCITRKGRRGGWCSNGVCRCTPN	0.724609	0.275391
Hill_BB_C8756	QPYQLQYEEDGPEYARELPIEEEELPSQVVEQHHQAKRATCDLLSPFKVGHAACVLDGFAMGRRGGWC	0.000000	1.000000
Hill_BB_C13793	KESSDPDSALYSDIHPRFRRQLPCDYLSGLGFGEDACNTDCIAKGHKSGFCTGLVCRCRTL	0.051485	0.948515
NHill_AD_C73537	GQSEASWWKKVFKPVEKLGQRVRDATIQGIGIAQQGANVLATVRGGPPQ	0.508308	0.491692
NHill_AD_C16493	GQSEAGWWKRVFKPVEKFGQRVRDAGVQGIAIAQQGANVLATARGGPPQQG	0.520865	0.479135
NHill_AD_C12927	GWWKRVFKPVEKLGQRVRDAGIQGLEIAQQGANVLATARGGPPQQG	0.389374	0.610626
NHill_AD_C12928	GWWKRVFKPVERLGQRVRDAGIQGLQIAQQGANVLATVRGGPPQQG	0.492318	0.507682
NHill_AD_C4669	SWFKKVFKPVEKVGQRVRDAGIQGVAIAQQGANVLATARGGPPH	0.901851	0.098149
Hill_BB_C3195	GWWKKVFKPVEKLGQRVRDAGIQGIAIAQQGANVLATVRGGPPQ	0.839903	0.160097
Hill_SB_C698	GQSEAGWWKRVFKPVEKFGQRVRDAGIQGIEIAQQGANVLATARGGPPQQG	0.519633	0.480367
Hill_SB_C2730	GWWKRVFKPVEKLGQRVRDAGIQGLEIAQQGANVLATVRGGPPQQG	0.481171	0.518829
Hill_SB_C1875	GQGESRSLWKKIFKPVEKLGQRVRDAGIQGIAIAQQGANVLATVRGGPPQ	0.702695	0.297305
Hill_BB_C5151	GQSESRSLWKKLFKPVERAGQRIRDATIKGIVIAQQGANVLATIRGGPAIPPGQG	0.870751	0.129249
Hill_BB_C390	FNNLPICVEGLAGDIGSILLGVESDIGALAGAIANLALIAGECAAQGEAGAAICA	0.908553	0.091447
NHill_AD_C53857	CINNGDGCQPDGRQGNCCSGYCHKEPGWVTGYCR	0.991593	0.008407
NHill_AD_C49215	CIANGNGCQPDGRQGNCCSGFCYKQRGWVAGYCRRR	0.994731	0.005269
Hill_BB_C2323	QLNIQGGGSPHSGFDLSVQGRAKIWESDNGRNTLYGTGQYGQHLGGPYGNSEPSFGGGLMFSHRF	0.071113	0.928887
Hill_BB_C7345	SIDDLTLSEDGEDHVEIITDDEVQRAKR	0.014171	0.985829
Hill_BB_C7346	QLNIQGGGSPHSGFDLNVQGRAKIWESNNGRNTLHGTGEYSQHLGGPYGNSRPNFGGGLLFTHRF	0.035845	0.964155
Hill_BB_C11803	QLNIQGGGSPHSGFNLSIQGQKKLWESNNKRNTLHGTGQYSQHFGGPYGNSRPNFGGGLVFTHRF	0.066283	0.933717
Hill_BB_C21232	QLNIQGGSKSTFLILISMSKVVRESNNGHETLHGTGDYNQHLGGPYGNSQPNFGGELLFTHRFKLCSLSKLLIVCVFSKCRK	0.945162	0.054838
NHill_AD_C17624	QIFAQGGGSPGKGYDIYAQGRAKLWESQNQRNSLHGTASYSQHLGGPYGNSRPNVGGGLTFTHRF	0.075412	0.924588
Hill_LB_C16634	IKCTASICTQICRILKYKCGYCASASRCVCLK	0.960433	0.039567
Hill_LB_C37730	AFAFDVTRKINPETSAVERPEVSEYPEIPKGTKLQEFVMMDIEIEEEGADNRAETIQRIKCVPSQCNQICRVLGKKCGYCKNASTCVCLG	0.006798	0.993202
Hill_BB_C46948	RKCTASQCTRVCKKLGYKRGYCQSSTKCVC	0.782932	0.217068
Hill_BB_C16137	MNIQGNAVSNPAGGQDVTVTAGKQFGSDNANITAGGFAGGNTLRGPPNAGVFASANANGHSLSVSKTVVPGISSTTSHGASANLFR	0.574294	0.425706
Hill_BB_C16883	QLSGSITPDMAGGNNVNIMASKFLGNPNHNIGGGVFASGNTRSNTPSLGAFGTLNLKDHSLGVSKTITPGVSDTFSQNARLIILKTPDHRVDANVFNSHTRLNNGFAFDKRGGSLDYTHRAGHSLSLGASHIPKFGTTAELTGKANLWKSPSGLSTFDLTGSAS	0.883543	0.116457
Hill_BB_C10074	SPQDGRRGSASVTVNNESRRGTDVRADLNARLWEGNNRRSSLDANAYYQRHFGGPMGTGRPDAGVGLNFRHRF	0.000017	0.999983
Hill_BB_C9237	MNIQGNAVSNPAGGQDVTVTAGKQFGSDNTNITAGAFAGGNTLRGPPNAGVFASANANSHSLSVSKTVVPGVSATTSHAASANLFRNDQHSVNAQAFSSATKLNDGFQFKQHGAGLNYNNANGHGASIGVNKIPGFGSSMDVGARANIFQNPNTSFDVMANSRTHLSGPFQGKTNFGVSAGITRRF	0.434155	0.565845
NHill_AD_C40487	MNIQGNAVSNPAGGQDVTVTAGKQFGSDNTNITAGAFAGGNTLRGPPNAGVFASANANGHSLSVSKTVVPGVSSTTSHAASANLFRNDQHNVNAQAFSSATKLNDGFQFKQHGAGLNYNNANGHGASIGVNKIPGFGSSMDVGARANIFQNPNTSFDVMANSRTHLSGPFQGKTNF	0.443017	0.556983
Hill_BB_C7758	AACDLFSALNVASSICAAHCLYLGYKGGYCDSKLVCVCR	0.791573	0.208427
Hill_BB_C14087	VTCDLLEPFLGPAPCMIHCIVRFRKRTGYCNSQNVCVCRG	0.391809	0.608191
Hill_LB_C29142	ATCDLLSPFKVGHAACAAHCIARGKRGGWCDKRAVCNCRK	0.450101	0.549899
Hill_BB_C308	VSCWFENENIKASACQMSCMYRKGRRGGMCVNGVCTCSPN	0.444002	0.555998
Hill_BB_C1619	LSCLFENQAVSAIACGSSCIARKGRRGGYCRNGVCVCTDN	0.954283	0.045717
Hill_BB_C1826	TTCTHLNCKLHCLLQRKRSGRCDRFNICKCIS	0.068550	0.931450
Hill_BB_C6571	ATCTNWNCRTQCIARGKRGGYCVERNICKCTS	0.842113	0.157887
Hill_BB_C7081	ATCDLISGTKIENVACAAHCIAMGHKGGYCNSNLICICR	0.945143	0.054857
Hill_BB_C7985	FTCSNLGCKAQCIILGNRSGGCNRLGVCQCN	0.822369	0.177631
Hill_BB_C7176	ATCDLLSPFKVGHAACALHCIALGRRGGWCDGRAVCNCRR	0.011073	0.988927
Hill_BB_C2519	ATCDLLSPFKVGHAACALHCIAMGRRGGWCDGRAVCNCRR	0.020927	0.979073
Hill_BB_C8473	ATCDLLSPFGVGHAACAVHCIAMGRRGGWCDDRAVCNCRR	0.165217	0.834783
Hill_BB_C34351	AMCDLLSGLNMGRSVCAMRCILKGHRGGWCDDQGVCNCRV	0.029224	0.970776
Hill_BB_C4683	RPDNIEYLEDSQVAELVRHKRLSCLFENEAISALACGASCITRKGRRGGWCSNGVCHCTPN	0.224878	0.775122
Hill_BB_C4977	LSCWFENEDIKATACAMSCIYRKGRKGGRCENGICRCTPN	0.106600	0.893400
Hill_BB_C13326	LSCLFENQAVSAIACGASCITRKGKRGGWCSNGVCRCTPN	0.701191	0.298809
Hill_BB_C7171	TTCDLISGTKIENIACAAHCIAMGHKGGYCNSNLICICR	0.952388	0.047612
Hill_BB_C10649	QFDNLEDTGVEEKVRHKRLTCLFDNRPISAFACGSNCVSRKGKRGGWCVNGVCRCT	0.974103	0.025897
Hill_BB_C13792	KQSSDPESALYSDIHPRFRRQLPCDYLSGLGFGEDACNTDCIAKGHKSGFCTGLVCRCRTL	0.295265	0.704735
Hill_BB_C15867	VTCDLLKPFFGRAPCMMHCILRFKKRTGFCSRQNVCVCR	0.182360	0.817640
NHill_AD_C69719	DVSIGSCVWGGSNYVSDCNGECKRRGYKGGHCGSFLNNICWCET	0.924393	0.075607
Hill_BB_C49430	APQFGGQIGGFGGGGFGGGGFGPGGGFRPGGVAEFQESSSSVNVERETFDQGGFEISDSSVTSSSVSESFRD	0.330011	0.669989

From left to right are shown in order: peptide contig, peptide sequence, the anticancer and non-anticancer scores related to each sequence.

**Table 3:** Results obtained with the AVPpred server for the antiviral activity prediction and with Antifp server for the antifungal activity prediction.

Peptide	AVPpred: antiviral activity prediction					Antifp: antifungal activity prediction	
	AVP motif (model)	Alignment model	Composition model	Physio-chemical model	Overall prediction	Score	Prediction
Hill_BB_C14202	–	Non-AVP	53.26	64.08	Yes	0.20892203	Non-antifungal
Hill_BB_C3566	–	Non-AVP	42.65	64.08	No	0.26737087	Non-antifungal
Hill_BB_C1152	–	Non-AVP	31.33	64.08	No	− 0.21250625	Non-antifungal
Hill_BB_C1153	–	Non-AVP	38.83	64.08	No	− 0.37506205	Non-ANTIFUNGAL
Hill_BB_C2676	–	Non-AVP	46.61	64.08	No	− 0.17216018	Non-antifungal
Hill_BB_C269	–	Non-AVP	52.07	64.08	Yes	− 0.025392142	Non-antifungal
Hill_BB_C1169	–	Non-AVP	44.47	64.08	No	0.072220496	Non-antifungal
Hill_BB_C779	–	Non-AVP	41.2	64.08	No	− 0.33302841	Non-antifungal
Hill_LB_C36111	–	Non-AVP	40.25	64.08	No	− 0.21911853	Non-antifungal
Hill_LB_C12085	–	Non-AVP	42.31	64.08	No	0.139426	Non-antifungal
Hill_BB_C1290	–	Non-AVP	31.53	64.08	No	0.11095482	Non-antifungal
Hill_BB_C7347	–	Non-AVP	39.24	64.12	No	− 0.040857298	Non-antifungal
Hill_BB_C9109	–	Non-AVP	23.7	64.08	No	− 0.068718526	Non-antifungal
Hill_BB_C11804	–	Non-AVP	39.24	64.12	No	− 0.040857298	Non-antifungal
Hill_BB_C309	–	Non-AVP	48.85	64.73	No	0.065455296	Non-antifungal
Hill_BB_C1827	–	Non-AVP	46.85	49.78	No	0.73998352	Antifungal
Hill_BB_C5878	Yes	Non-AVP	50.55	67.39	Yes	− 0.16644401	Non-antifungal
Hill_BB_C8756	–	Non-AVP	26.31	64.08	No	− 0.34776804	Non-antifungal
Hill_BB_C13793	–	Non-AVP	42.66	64.09	No	0.25709331	Non-antifungal
NHill_AD_C73537	–	Non-AVP	33.7	63.94	No	− 0.36753515	Non-antifungal
NHill_AD_C16493	–	Non-AVP	34.66	64.07	No	− 0.43908213	Non-antifungal
NHill_AD_C12927	–	Non-AVP	39.89	64.07	No	− 0.47185039	Non-antifungal
NHill_AD_C12928	–	Non-AVP	40.33	64.09	No	− 0.40020762	Non-antifungal
NHill_AD_C4669	–	Non-AVP	36.71	63.87	No	− 0.031971647	Non-antifungal
Hill_BB_C3195	–	Non-AVP	37.43	64.08	No	− 0.24406508	Non-antifungal
Hill_SB_C698	–	Non-AVP	33.23	64.07	No	− 0.43908213	Non-antifungal
Hill_SB_C2730	–	Non-AVP	39.88	64.09	No	− 0.38062322	Non-antifungal
Hill_SB_C1875	–	Non-AVP	34.95	63.96	No	− 0.22572859	Non-antifungal
Hill_BB_C5151	–	Non-AVP	31.71	64.03	No	− 0.34876968	Non-antifungal
Hill_BB_C390	–	Non-AVP	52.45	64.08	Yes	− 0.67921544	Non-antifungal
NHill_AD_C53857	–	Non-AVP	51.96	65.69	Yes	0.12385895	Non-antifungal
NHill_AD_C49215	–	Non-AVP	46.35	65.52	No	0.2406468	Non-antifungal
Hill_BB_C2323	–	Non-AVP	19.92	64.08	No	− 0.10977439	Non-antifungal
Hill_BB_C7345	–	Non-AVP	26.56	47.85	No	− 0.87408278	Non-antifungal
Hill_BB_C7346	–	Non-AVP	23.75	64.08	No	− 0.059453989	Non-antifungal
Hill_BB_C11803	–	Non-AVP	28.99	64.08	No	− 0.052337869	Non-antifungal
Hill_BB_C21232	–	Non-AVP	44.14	64.08	No	− 0.070217673	Non-antifungal
NHill_AD_C17624	–	Non-AVP	23.01	64.08	No	− 0.15660532	Non-antifungal
Hill_LB_C16634	–	Non-AVP	53.29	64.88	Yes	0.7067461	Antifungal
Hill_LB_C37730	–	Non-AVP	34.85	64.08	No	0.38202837	Non-antifungal
Hill_BB_C46948	–	Non-AVP	48.59	64.22	No	0.71418843	antifungal
Hill_BB_C16137	–	Non-AVP	28.08	64.08	No	0.010457995	Non-antifungal
Hill_BB_C16883	–	Non-AVP	25.14	64.08	No	− 0.52680116	Non-antifungal
Hill_BB_C10074	–	Non-AVP	12.45	64.08	No	− 0.19881079	Non-antifungal
Hill_BB_C9237	–	Non-AVP	28.71	64.08	No	0.32515345	Non-antifungal
NHill_AD_C40487	–	Non-AVP	28.54	64.08	No	0.37181457	Non-antifungal
Hill_BB_C7758	–	Non-AVP	61.82	64.18	Yes	0.18741319	Non-antifungal
Hill_BB_C14087	Yes	Non-AVP	63.07	66.59	Yes	0.10302883	Non-antifungal
Hill_LB_C29142	–	Non-AVP	52.07	64.12	Yes	0.33363813	Non-antifungal
Hill_BB_C308	–	Non-AVP	48.85	64.73	No	0.065455296	Non-antifungal
Hill_BB_C1619	Yes	Non-AVP	52.47	68.2	Yes	− 0.12761437	Non-antifungal
Hill_BB_C1826	–	Non-AVP	46.42	49.91	No	0.2129187	Non-antifungal
Hill_BB_C6571	–	Non-AVP	49.54	67	No	0.5009657	Antifungal
Hill_BB_C7081	–	Non-AVP	51.03	64.65	Yes	0.35232096	Non-antifungal
Hill_BB_C7985	–	Non-AVP	48.06	65.99	No	0.44711187	Non-antifungal
Hill_BB_C7176	–	Non-AVP	55.82	64.95	Yes	0.27115344	Non-antifungal
Hill_BB_C2519	–	Non-AVP	53.4	64.85	Yes	0.27115344	Non-antifungal
Hill_BB_C8473	–	Non-AVP	47.7	64.69	No	0.21172458	Non-antifungal
Hill_BB_C34351	–	Non-AVP	50.25	64.13	Yes	0.10334371	Non-antifungal
Hill_BB_C4683	–	Non-AVP	39.94	64.09	No	− 0.25553273	Non-antifungal
Hill_BB_C4977	–	Non-AVP	52.52	65.92	Yes	0.0078493215	Non-antifungal
Hill_BB_C13326	Yes	Non-AVP	56.26	68.51	Yes	− 0.21725812	Non-antifungal
Hill_BB_C7171	–	Non-AVP	44.07	64.19	No	0.21225639	Non-antifungal
Hill_BB_C10649	–	Non-AVP	45.72	64.11	No	− 0.13179766	Non-antifungal
Hill_BB_C13792	–	Non-AVP	47.38	64.08	No	1.0166485	Antifungal
Hill_BB_C15867	–	Non-AVP	66.1	63.61	Yes	0.70687492	Antifungal
NHill_AD_C69719	Yes	Non-AVP	47.27	64.08	Yes	0.91354184	Antifungal
Hill_BB_C49430	–	Non-AVP	33.03	64.08	No	− 0.36274044	Non-antifungal

From left to right are shown in order: peptide contig, AVP motif model results, alignment model results, composition model results, the physio-chemical model results, the overall results for the antiviral prediction, antifungal score and prediction result for the antifungal activity.

### Physicochemical properties of the identified peptides

**Figure 1:**
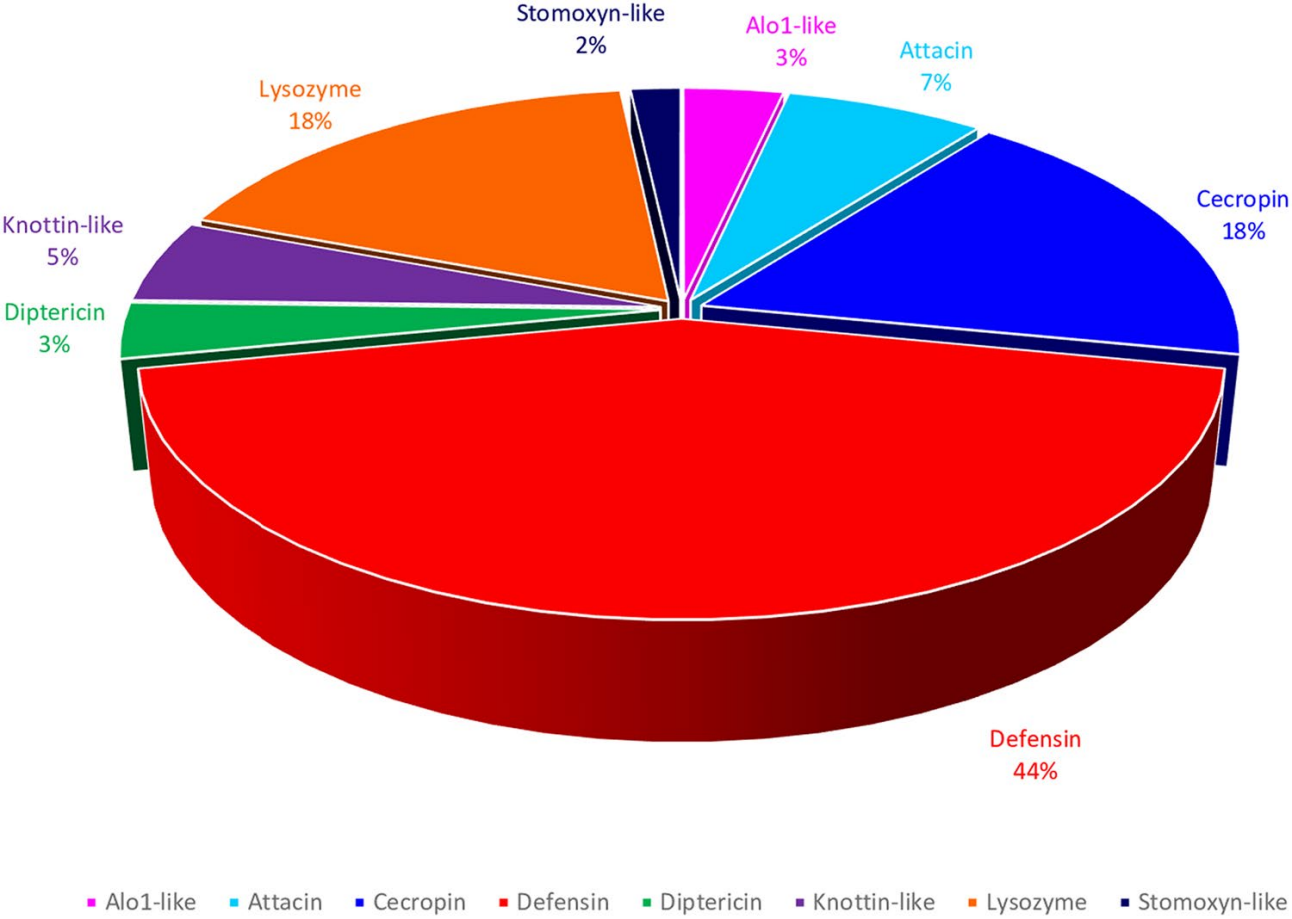
Graphic representation of the identified AMP classes from larvae and adult transcriptomes. The pie chart shows that the largest number of identified peptides belongs to the class of defensins.

**Figure 2:**
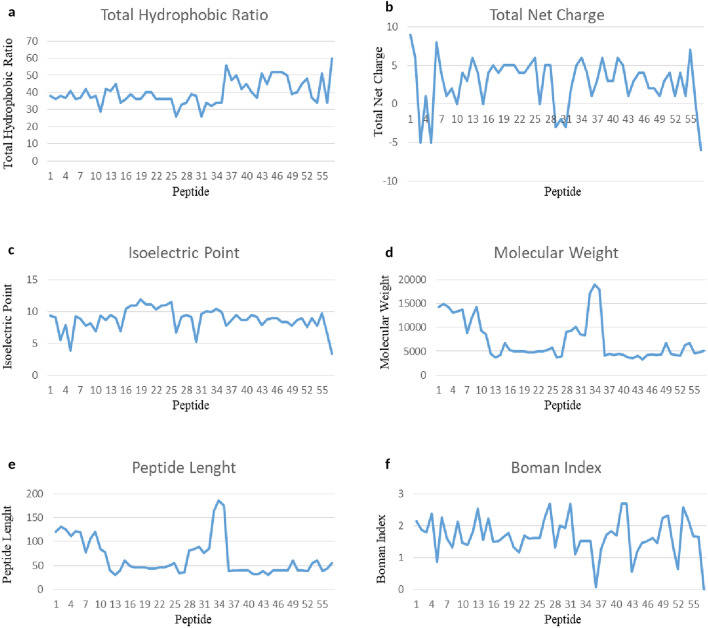
Graphical representation of the physicochemical properties of the 57 peptides with putative activity: (**a**) total hydrophobic ratio; (**b**) total net charge; (**c**) isoelectric point; (**d**) molecular weight; (**e**) peptide length; (**f**) Boman Index.

The 57 identified, putatively active, peptides belong to different classes of AMPs including defensins, cecropins, attacins and lysozyme (Fig. 1). Although attacins and lysozyme are proteins due to their high molecular weight, they belong to AMPs’ classes because of their antibacterial activity. The physicochemical properties of these peptides were evaluated with the Antimicrobial Peptide Database Calculator and Predictor APD3 (Table 4). Figure 2 shows the graphical representation of the calculated physicochemical properties of the 57 identified peptides, whereas Table 5 reports their amino acid composition and the amino acid frequency, compared to the amino acid composition of the patent AMPs available in the APD database. The highest amino acid content in all the analysed AMPs was found for Gly, Ala, Arg, Asn, Cys, Leu, Ser residues, whereas the lowest content was found for His, Met, Trp, Tyr residues (Table 5). A graphical representation of the amino acid composition of each identified peptide is shown in Supplementary Fig. 1. The molecular mass of the identified peptides ranges from 3000 Da for the smallest peptide Hill_BB_C7985 to 19,000 Da for the largest peptide Hill_BB_C9237, with an average of approximately 7000 Da. The amino acid sequences varied from a minimum value of 31 residues to a maximum of 186 residues, and an average of approximately 66 residues. The total hydrophobic ratio showed the lowest value of 26 for the peptide NHill_AD_C53857 and the highest of 60 for the peptide Hill_BB_C390, and an average value of approximately 40. The total net charge of the identified peptides ranged from − 6, for the Hill_BB_C390 peptide to + 9 for the Hill_BB_C14202 peptide, with an average value of + 3, while the Isoelectric Point (pI) varied from 3.34 for the Hill_BB_C390 peptide to 11.83 for the NHill_AD_C12928 peptide, with an average value of 8.79.

**Table 4:** Prediction of physicochemical properties using the Antimicrobial Peptide Database Calculator and Predictor (APD3) and the Compute pI/Mw tool—Expasy.

Peptide	Lenght (aa)	Molecular weight (g/mol)	Total hydrophobic Ratio (%)	Total net charge	pI	Boman Index (kcal/mol)
Hill_BB_C14202	121	14,282.443	38	+ 9	9.32	2.14
Hill_BB_C3566	131	14,871.993	36	+ 6	8.99	1.87
Hill_BB_C1152	126	14,259.799	38	− 5	5.55	1.8
Hill_BB_C1153	112	13,084.607	37	+ 1	7.84	2.37
Hill_BB_C2676	122	13,394.838	41	− 5	3.80	0.88
Hill_BB_C269	119	13,730.8	36	+ 8	9.24	2.26
Hill_BB_C1169	77	8763.954	37	+ 4	8.80	1.59
Hill_BB_C779	107	12,074.699	42	+ 1	7.76	1.32
Hill_LB_C36111	121	14,214.145	37	+ 2	8.15	2.13
Hill_LB_C12085	84	9307.51	38	0	6.88	1.45
Hill_BB_C1290	77	8480.598	29	+ 4	9.30	1.39
Hill_BB_C309	40	4422.19	42	+ 3	8.67	1.83
Hill_BB_C1827	31	3686.457	41	+ 6	9.38	2.53
Hill_BB_C5878	40	4204.904	45	+ 4	8.98	1.56
Hill_BB_C13793	61	6712.597	34	0	6.88	2.22
NHill_AD_C73537	49	5259.014	36	+ 4	10.43	1.49
NHill_AD_C16493	51	5404.099	37	+ 4	10.93	1.63
NHill_AD_C12927	46	4969.69	36	+ 4	10.93	1.65
NHill_AD_C12928	46	5024.777	36	+ 5	11.83	1.78
NHill_AD_C4669	44	4670.398	40	+ 5	11.07	1.32
Hill_BB_C3195	44	4726.506	40	+ 5	11.07	1.16
Hill_SB_C698	51	5476.163	35	+ 3	10.26	1.78
Hill_SB_C2730	46	4997.744	36	+ 4	10.93	1.60
Hill_SB_C1875	50	5312.123	36	+ 5	11.00	1.61
Hill_BB_C5151	55	5823.746	36	+ 6	11.47	1.61
NHill_AD_C53857	34	3679.079	26	0	6.70	2.23
NHill_AD_C49215	36	3985.541	33	+ 5	9.18	2.69
Hill_BB_C21232	82	9053.427	34	+ 5	9.46	1.32
Hill_LB_C16634	32	3531.395	53	+ 6	9.18	0.75
Hill_LB_C37730	90	10,059.611	38	− 2	5.17	1.93
Hill_BB_C46948	30	3390.071	33	+ 8	9.64	2.58
Hill_BB_C16137	86	8328.081	34	+ 2	9.98	1.11
Hill_BB_C16883	164	17,080.992	32	+ 5	9.89	1.51
Hill_BB_C9237	186	18,942.725	34	+ 6	10.36	1.52
NHill_AD_C40487	176	17,910.512	34	+ 4	9.87	1.52
Hill_BB_C7758	39	4089.842	56	+ 1	7.81	0.07
Hill_BB_C14087	40	4501.427	47	+ 3	8.69	1.28
Hill_LB_C29142	40	4275.055	50	+ 6	9.38	1.72
Hill_BB_C308	40	4422.19	42	+ 3	8.67	1.83
Hill_BB_C1619	40	4183.84	45	+ 3	8.69	1.7
Hill_BB_C1826	32	3752.517	40	+ 6	9.43	2.7
Hill_BB_C6571	32	3597.19	37	+ 5	9.18	2.7
Hill_BB_C7081	39	4055.809	51	+ 1	7.83	0.55
Hill_BB_C7985	31	3233.819	45	+ 3	8.70	1.18
Hill_BB_C7176	40	4259.049	52	+ 4	8.98	1.45
Hill_BB_C2519	40	4277.088	52	+ 4	8.98	1.52
Hill_BB_C8473	40	4249.98	52	+ 2	8.37	1.62
Hill_BB_C34351	40	4330.189	50	+ 2	8.36	1.46
Hill_BB_C4683	61	6736.674	39	+ 1	7.79	2.25
Hill_BB_C4977	40	4486.229	40	+ 3	8.66	2.33
Hill_BB_C13326	40	4162.859	45	+ 4	8.96	1.35
Hill_BB_C7171	39	4099.862	48	+ 1	7.54	0.64
Hill_BB_C10649	56	6263.181	37	+ 4	7.54	2.58
Hill_BB_C13792	61	6725.639	34	+ 1	7.78	2.17
Hill_BB_C15867	39	4566.65	51	+ 7	9.69	1.68
NHill_AD_C69719	44	4763.313	34	+ 1	6.71	1.66
Hill_BB_C390	55	5182.986	60	− 6	4.06	-

From left to right are shown in order: peptide contig, the peptide length, the molecular weight, the total hydrophobic ratio, the total net charge, the isoelectric point (pI) and the Boman index.

**Table 5:** Amino acid frequency and amino acid composition of the identified peptides.

Amino acid composition of peptides identified in *Hermetia illucens*			Amino acid composition of patent AMPs in the APD database
Amino acid three letter code	Amino acid frequency	Amino acid composition (%)	Amino acid composition (%)
Ala	297	7.98816	7.61
Arg	230	6.18612	5.81
Asn	258	6.93921	3.85
Asp	142	3.81926	2.65
Cys	262	7.04679	6.86
Glu	113	3.03927	2.69
Gln	166	4.46477	2.57
Gly	406	10.91985	11.56
His	89	2.39376	2.16
Ile	175	4.70683	5.93
Leu	242	6.50888	8.34
Lys	205	5.51372	9.55
Met	42	1.12964	1.25
Phe	143	3.84615	4.08
Pro	124	3.33513	4.69
Ser	270	7.26197	6.07
Thr	168	4.51856	4.51
Trp	85	2.28617	1.64
Tyr	85	2.28617	2.48
Val	216	5.80957	5.7
Total	3718	100	100

As it is shown, the Gly, Ala, Arg, Asn, Cys, Leu, Ser residues are the most abundant, whereas the lowest content is associated with the His, Met, Trp, Tyr residues.

### Bacterial cell growth and viability

Four putative antimicrobial peptides, namely Hill_BB_C6571, Hill_BB_C16634, Hill_BB_C46948 and Hill_BB_C7985, that showed high antimicrobial score values with all prediction softwares were selected and chemically synthesised. The antimicrobial activity of these peptides was verified by monitoring *E. coli* cells growth in the presence of different concentrations of each peptide in comparison with untreated cells. Supplementary Fig. 2 shows the growth curves of *E. coli* cells in the presence of 3 µM (A) or 12 µM (B) concentrations of each peptide. A clear decrease in the growth curves was observed at both concentrations compared to untreated cells (blue line) with bacteria impaired to achieve the exponential phase at 12 µM due to rapid death. The reduction in cell viability was observed with increasing concentration of each peptide in comparison with untreated cells.

Next, cell viability of *E. coli* was also evaluated by treatment with 3 µM of each peptide (Supplementary Fig. 2C) confirming a decrease of about 50% in cell viability after 100 min treatment with all four peptides analysed.

## Discussion

AMPs are promising candidates as alternatives to conventional antibiotics, thanks to their low toxicity to eukaryotic cells and their broad spectrum of action against bacteria, mycobacteria, fungi, viruses and cancer cells^24^. AMPs can kill bacteria through different mechanisms including membrane disruption, targeting intracellular components, or interfering with the bacterial metabolism^25–27^. Furthermore, most AMPs are cationic, with the positive net charge promoting the electrostatic interaction with negatively charged bacterial membranes^28^.

**Figure 3:**
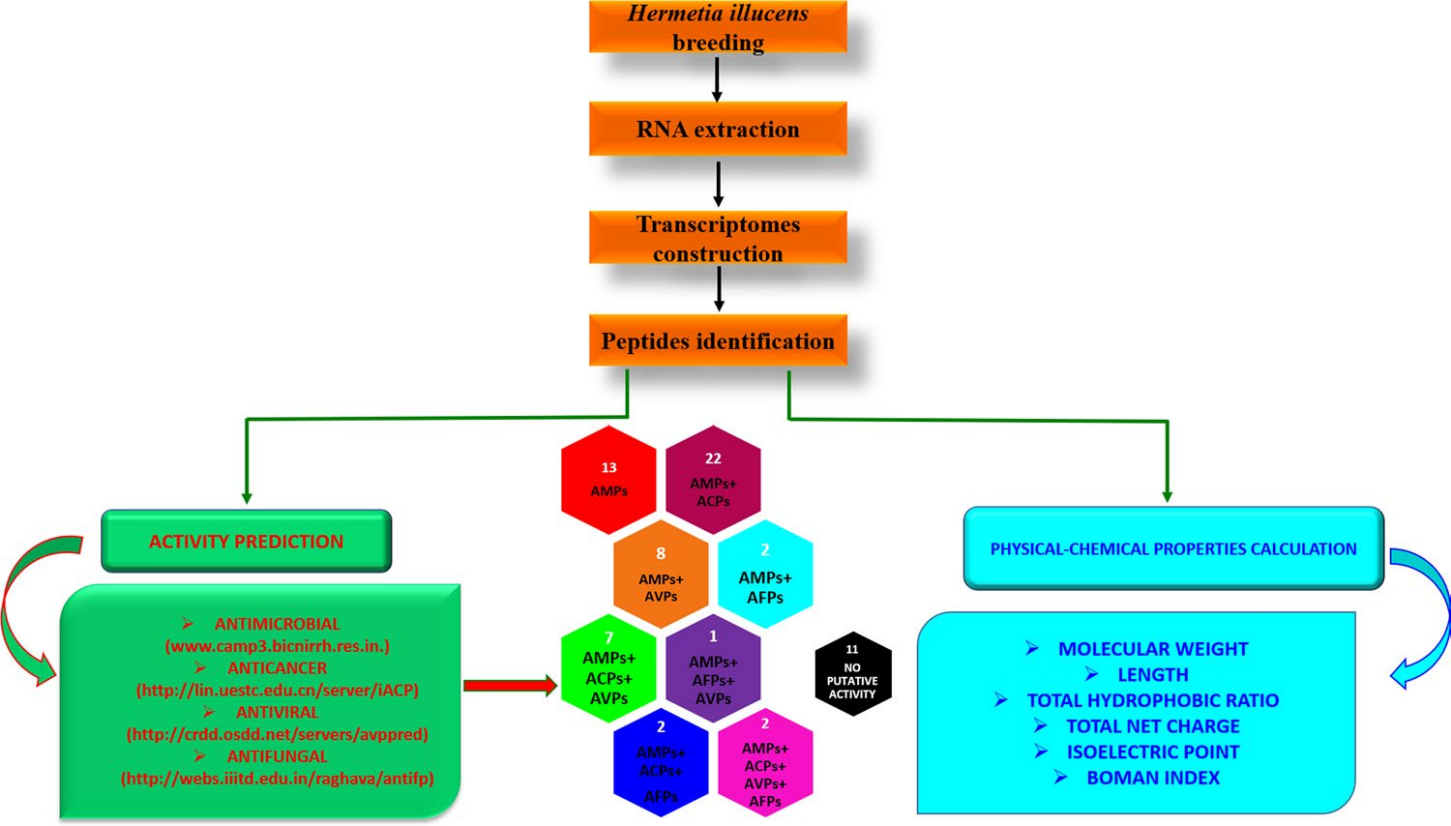
Strategies carried out in order to identify peptides from *Hermetia illucens* insect.

All living organisms produce AMPs with insects being among the richest sources due to their high biodiversity and their extremely varied living environments. The immune system of the insect *H. illucens* is very developed, as this species feeds on decaying substrates and manure, which are extremely rich in pathogenic microorganisms, as it possible to observe also in other species, such as in *Eristalis tenax*. Twenty-two AMPs were indeed identified in the Diptera *E. tenax*, that has been able to adapt to different aquatic habitats (sewage tanks and manure pits) with heavy microbial load^29^. AMPs, which are synthesized by the fat body and hemocytes and then secreted into the hemolymph, are an essential part of the immune defense^30, 31^. In this study, we focused on the gene level in order to identify all putative genes encoding AMPs (Fig. 3).

The transcriptomes of *H. illucens* larvae as well as the combined male and female adults were assembled, and all the obtained contigs were functionally annotated through the Blast2Go software resulting in the identification of 68 putative peptides of interest. These sequences were analyzed in silico through the CAMP database and the iACP online tool in order to evaluate their antimicrobial and anticancer activity, respectively. Additionally, the AVPpred and the Antifp servers were used to predict the antiviral and the antifungal activity, respectively, of the identified peptides. Our results led to the identification of 57 peptides, 13 of which were predicted as endowed with an antimicrobial activity, 22 with an antimicrobial and anticancer activity, eight with an antimicrobial and antiviral activity, two with an antimicrobial and antifungal activity, seven with an antimicrobial, anticancer and antiviral activity (Supplementary Table S1). Only one peptide was predicted as antimicrobial, antiviral and antifungal activity, whereas two peptides were predicted to have a putative antimicrobial, anticancer and antifungal activity (Supplementary Table S1). Surprisingly, two peptides, corresponding to Hill_LB_C16634 and NHill_AD_C69719 contigs, resulted positive to all activity predictions (Supplementary Table S1). Most of the identified peptides belong to defensins and cecropins families, whose composition ranges from 34 to 51 amino acids^32, 33^. These peptides have a pattern of six cysteines, which are involved in the formation of three disulphide bonds, Cys1–Cys4, Cys2–Cys5 and Cys3–Cys6, for insect defensins^34^. Insect defensins are active against Gram–negative bacteria such as *Escherichia coli*, but mainly against Gram-positive bacteria, such as *Staphylococcus aureus*, *Micrococcus luteus*, *Bacillus subtilis*, *Bacillus thuringiensis*, *Aerococcus viridians* and *Bacillus megaterium*. Moreover, some insect defensins are also active against fungi^35–39^. For example, the royalisin peptide, isolated from the royal jelly of *Apis mellifera*, consists of 51 amino acids, and the six cysteine residues are involved in the formation of three disulphide bonds and are active against Gram-positive bacteria and fungi^40^. Defensin targets have not been identified yet, and studies of the structure–activity relationship could be useful to understand the molecular mechanism underlying their bioactivity^41^.

Cecropins were first purified from the moth *H. cecropia* and represent the most abundant family of linear α-helical AMPs in insects, active against both Gram-negative and Gram-positive bacteria^42^. Insect cecropins, mainly derived from lepidopteran and dipteran species, are the cecropins A, B and D. These consist of 35–37 amino acids with no cysteine residues and are able to lyse the bacterial membrane and to reduce the proline uptake. For example, cecropin B, a linear cationic peptide consisting of 35 amino acids, reduces the lethality of *E. coli* load and plasma endotoxin levels, and also shows an antifungal activity against *Candida albicans*^42,43^. Moreover, a cecropin-like peptide was isolated from the salivary glands of the female mosquito *Aedes egypti*, showing antiviral activity against the Dengue virus. Glycine residue is the most spread among the peptides that we identified and is particularly related to Attacin proteins^44,45^. Although the mechanism of action of the different AMPs has not yet been fully elucidated, it appears that AMPs, unlike antibiotics, have more difficulty in causing a microbial resistance, and most of them do not destroy normal cells of higher animals^46^. Recently, it has been demonstrated that the clavaspirin peptide from tunicate *Styela clava* exhibits the ability to kill drug-resistant pathogens, such as *S. aureus*, without a detectable resistance^47^. Moreover, it was demonstrated that two proline rich peptides (Lser-PRP2 and Lser-PRP3) do not interfere with protein synthesis but both were able to bind the bacterial chaperone DnaK and are therefore able to inhibit protein folding^48^. The characteristics of AMPs make them excellent candidates for the development of new drugs.

The bioinformatic approach represents a powerful tool to predict the physicochemical properties and the putative function of amino acid sequences. However, we aimed to go beyond the simple functional annotation which typically exclusively relies on sequence similarities to peptides deposited in public databases. Indeed, the approach we reported is based on the use of several softwares, previously employed to perform similar analyses^49–51^, that exploit different algorithms for the determination of a score that predicts the biological activity of unknown peptides. We demonstrated that a similar approach can provide reliable indications about the potential biological activities of candidate AMPs, as confirmed by our preliminary tests on the antimicrobial activity of four identified AMPs (Supplementary Fig. 2). However, validation studies were out of the scope of this study which was essentially aimed to identify a set of candidate peptides which could serve as a starting point for subsequent functional characterization of *H. illucens* AMPs by our group, as well as by other researchers in the field**.** Indeed, following the in silico analysis, the largest peptides could be produced by recombinant methodologies while chemical synthesis could be used for smaller ones. Structural analysis could be performed through mass spectrometry and circular dichroism (CD) and the biological activity could be evaluated by in vitro tests. The produced peptides, in fact, could be tested in vitro to validate their activity against different bacterial strains, both Gram-negative and Gram-positive bacteria, cancer cell lines, and fungi. Moreover, the peptides showing interesting biological activities, could be produced in fusion with suitable tags to investigate their mechanism of action through functional proteomics experiments and advanced mass spectrometry methodologies, in order to characterise their interaction(s) with target protein (mainly components of the biological membranes), thus identifying the possible protein targets.

## Materials and methods

### Rearing of Hermetia illucens and RNA isolation

*Hermetia illucens* larvae were reared on different diets in order to minimize the possible effect of a specific substrate on the expression of peptides, according to the protocol adopted by Vogel et al.^52^. The adults were reared in an environmental chamber under controlled conditions: temperature 27 ± 1.0 °C, humidity 70% ± 5%, and a photoperiod of 12:12 h [L:D]. Since it is not clear whether all AMPs are expressed in a similar fashion across different larval instars, RNA was obtained from two different instars, in order to identify the maximum number of expressed AMPs. Thus, using the TRI Reagent following the manufacturer’s instructions (Sigma, St. Louis, Missouri, USA), RNA was extracted from adults’ total body and from two larval stages: 2^nd^ and 5^th^ instar larvae whose isolated RNA was subsequently pooled in a 1:1 ratio for RNAseq. A DNase (Turbo DNase, Ambion Austin, Texas, USA) treatment was carried out to eliminate any contaminating DNA. After the DNase enzyme removal, the RNA was further purified using the RNeasy MinElute Clean up Kit (Qiagen, Venlo, Netherlands) following the manufacturer’s protocol, and eluted in 20 μL of RNA Storage Solution (Ambion Austin, Texas, USA). The RNA integrity was verified on an Agilent 2100 Bioanalyzer using the RNA Nano chips (Agilent Technologies, Palo Alto, CA), and the RNA quantity was determined by a Nanodrop ND1000 spectrophotometer.

### *RNA-Seq,* de novo *larvae and combined adult male and female transcriptomes assembly and gene identification*

The transcriptome sequencing of all RNA samples was performed with a poly(A) + enriched mRNA fragmented to an average of 150 nucleotides. The sequencing was carried out by the Max Planck Genome Center (https://mpgc.mpipz.mpg.de/home/) using standard TruSeq procedures on an Illumina HiSeq2500 sequencer. The de novo transcriptome assembly was carried out using a CLC Genomics Workbench v7.1 (https://www.clcbio.com) which is designed to assemble large transcriptomes using sequences from short-read sequencing platforms. All obtained sequences (contigs) were used as queries for a BLASTX search^53^ in the ‘National Center for Biotechnology Information’ (NCBI) non-redundant (nr) database, considering all hits with an E-value cut-off of 10^–5^. The transcriptomes were annotated using BLAST, Gene Ontology, and InterProScan searches using Blast2GO PRO v2.6.1 (https://www.blast2go.de)^54^. To optimize the annotation of the obtained data, GO slim was used, a subset of GO terms that provides a higher level of annotations and allows a more global view of the result. Candidate AMP genes were identified through an established reference set of insect-derived AMPs and lysozymes, and additional filtering steps to avoid interpreting incomplete genes or allelic variants as further AMP genes^52^.

### In silico *analysis for the antimicrobial, anticancer, antiviral and antifungal activity prediction*

The sequences, functionally annotated as antimicrobial peptides by the Blast2Go software, were analysed with Prop 1.0^55^ and Signal P 4.0^56^ Servers in order to identify the signal peptide and the pro-peptide region. The mature and active peptide regions were analysed in silico by four machine-learning algorithms, available on the CAMP database^57^: Support Vector Machine (SVM), Discriminant Analysis (DA), Artificial Neural Network (ANN), and Random Forest (RF), in order to predict their antimicrobial activity. The minimum calculated threshold for a sequence in order to be considered antimicrobial is 0.5^67–69^. When all the sequences were analyzed with the algorithms, the ones with a score higher than 0.5 were automatically considered putative antimicrobials by the software. We would like to point out that the threshold is intrinsically set by the software, and can’t be modified by the user. This is true for the SVM, RF and DA algorithms that report the result in a numerical form (score) while the ANN algorithm provides the results as categories, namely either AMP (antimicrobial) or NAMP (not-antimicrobial). All sequences that showed a positive result with all four statistical methods, were considered as antimicrobial. The iACP tool^58–62^ was used to predict the anticancer activity of the same sequences, providing the results in a numerical form. The prediction of the antiviral activity was performed in silico with the online server AVPpred. It exploits four different models: (1) the AVP motif, which returns the result as YES or NO; (2) the Alignment model, which gives the result in the form AVP or Non-AVP; (3) the Composition model and the (4) the Physico-chemical model, which return their results in a numerical form (percentage). The overall result is expressed with a YES, if the peptide results have a putative antiviral activity, and with a NO, if otherwise^63^. The Antifp server was used to predict the antifungal activity, and provides the result as a numerical score^64^. For this analysis, a threshold of 0.5 was used.

### Evaluation of the physicochemical properties

The corresponding physicochemical properties of identified putative active peptides following an in silico analysis, such as peptide length, molecular weight, total hydrophobic ratio, total net charge, isoelectric point, and the Boman Index, were determined by the Antimicrobial Peptide Database Calculator and Predictor (APD3)^65–67^ and the Compute pI/Mw tool—Expasy^68, 69^.

### Bacterial cell growth and viability

Four putative antimicrobial peptides, namely Hill_BB_C6571, Hill_BB_C16634, Hill_BB_C46948 and Hill_BB_C7985, that showed high antimicrobial score values with all prediction softwares were selected and chemically synthesised (Bio-Fab Research, Rome, Italy). *E. coli* cells were incubated overnight in LB medium at 37 °C. The culture was then diluted to a concentration of 0.08 OD_600_/mL in fresh medium and grown at 37 °C for 90 min. At an OD/mL value of 0.5, the antimicrobial peptides were added to the culture at a final concentration of 3 or 12 µM. Growth of the culture was evaluated every 20 min for a total of 120 min by assessing absorbance at 600 nm.

Cell viability was evaluated by enumerating Colony Forming Units (CFU) after 16 h of incubation with 3 µM of each peptide. Serial dilutions of bacterial cultures up to a concentration of 10^–6^ cells both for treated and untreated samples were prepared. Finally, 100 µL of each sample was plated on LB agar every 20 min for a total of 100 min. Plates were incubated for 16 h at 37 °C and the CFUs occurring on each plate were then counted. Experiments were performed in triplicate.

## Supplementary information

Supplementary Information.
