# Supplementary material for: A bioinformatic study of antimicrobial peptides identified in the Black Soldier Fly (BSF) Hermetia illucens (Diptera: Stratiomyidae)
Source: Sci Rep. 2020 Oct 9;10:16875. doi: 10.1038/s41598-020-74017-9 (PMC7547115; doi:10.1038/s41598-020-74017-9)
Supplement: Supplementary file 1 — Supplementary Information. [file 41598_2020_74017_MOESM1_ESM.docx]

A Bioinformatic Study of Antimicrobial Peptides Identified in the Black Soldier Fly (BSF) *Hermetia illucens* (Diptera: Stratiomyidae)

**Antonio Moretta^1^, Rosanna Salvia^1^, Carmen Scieuzo^1^, Angela Di Somma^2^, Heiko Vogel^3^, Pietro Pucci^4^, Alessandro Sgambato^5,6^, Michael Wolff^7^, Patrizia Falabella*^1^**

**1 Department of Sciences, University of Basilicata, Via dell'Ateneo Lucano 10, 85100, Potenza, Italy.**

**2 Department of Chemical Sciences, University Federico II of Napoli, Via Cinthia 6, 80126 Napoli. Italy**

**3 Department of Entomology, Max Planck Institute for Chemical Ecology, Hans-Knöll-Straße 8, D-07745, Jena, Germany.**

**4 CEINGE Advanced Biotechnology, Via Gaetano Salvatore 486, Naples, Italy.**

**5 Centro di Riferimento Oncologico della Basilicata (IRCCS-CROB), Rionero in Vulture (PZ), Italy;**

**6 Department of Translational Medicine and Surgery, Università Cattolica del Sacro Cuore, Rome, Italy.**

**7 Institute of Bioprocess Engineering and Pharmaceutical Technology, Technische Hochschule Mittelhessen, Wiesenstrasse 14, 35390 Giessen, Germany.**

***** Corresponding author: patrizia.falabell@unibas.it

| **PREDICTED ACTIVITY** | **PEPTIDE** |
| --- | --- |
| AMPs | Hill_BB_C1153  Hill_BB_C309  Hill_BB_C13793  NHill_AD_C12927  NHill_AD_C12928  Hill_SB_C2730  Hill_LB_C37730  Hill_BB_C9237  NHill_AD_C40487  Hill_BB_C308  Hill_BB_C1826  Hill_BB_C8473  Hill_BB_C4683 |
| AMPs + ACPs | Hill_BB_C3566  Hill_BB_C1152  Hill_BB_C2676  Hill_BB_C1169  Hill_BB_C779  Hill_LB_C36111  Hill_LB_C12085  Hill_BB_C1290  NHill_AD_C73537  NHill_AD_C16493  NHill_AD_C4669  Hill_BB_C16137  Hill_BB_C3195  Hill_SB_C698  Hill_SB_C1875  Hill_BB_C5151  NHill_AD_C49215  Hill_BB_C21232  Hill_BB_C16883  Hill_BB_C7985  Hill_BB_C7171  Hill_BB_C10649 |
| AMPs + AVPs | Hill_BB_C14202  Hill_BB_C269  Hill_BB_C14087  Hill_LB_C29142  Hill_BB_C7176  Hill_BB_C2519  Hill_BB_C34351  Hill_BB_C4977 |
| AMPs + AFPs | Hill_BB_C1827  Hill_BB_C13792 |
| AMPs + ACPs + AVPs | Hill_BB_C5878  Hill_BB_C390  NHill_AD_C53857  Hill_BB_C7758  Hill_BB_C1619  Hill_BB_C7081  Hill_BB_C13326 |
| AMPs + AFPs + AVPs | Hill_BB_C15867 |
| AMPs + ACPs + AFPs | Hill_BB_C46948  Hill_BB_C6571 |
| AMPs + ACPs + AVPs + AFPs | Hill_LB_C16634  NHill_AD_C69719 |

**Supplementary Table S1. Summary of all activities predicted for the identified peptides.** The acronyms AMPs, ACPs, AVPs and AFPs represent antimicrobial, anticancer, antiviral, and antifungal, respectively.

**
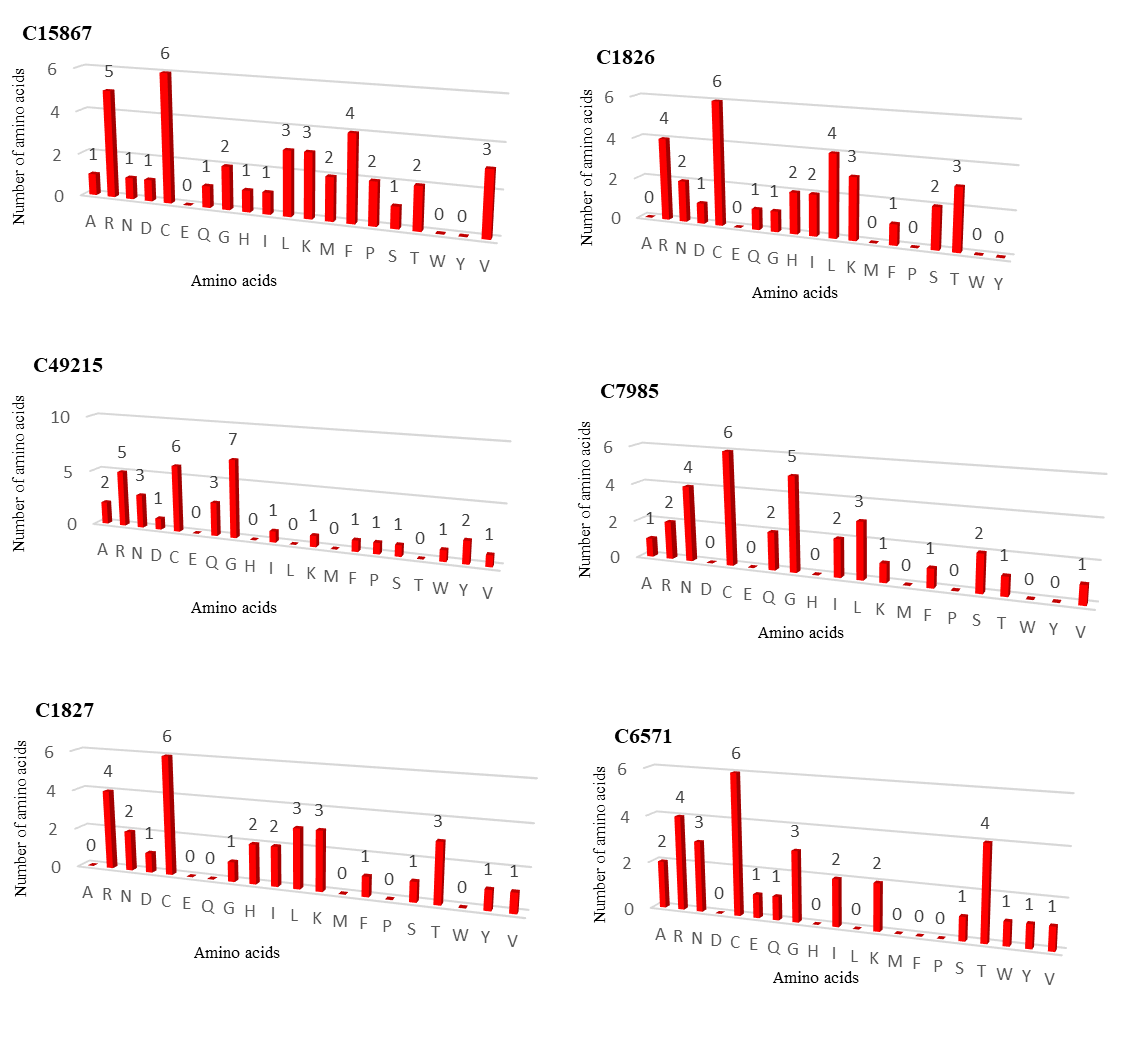
**

**
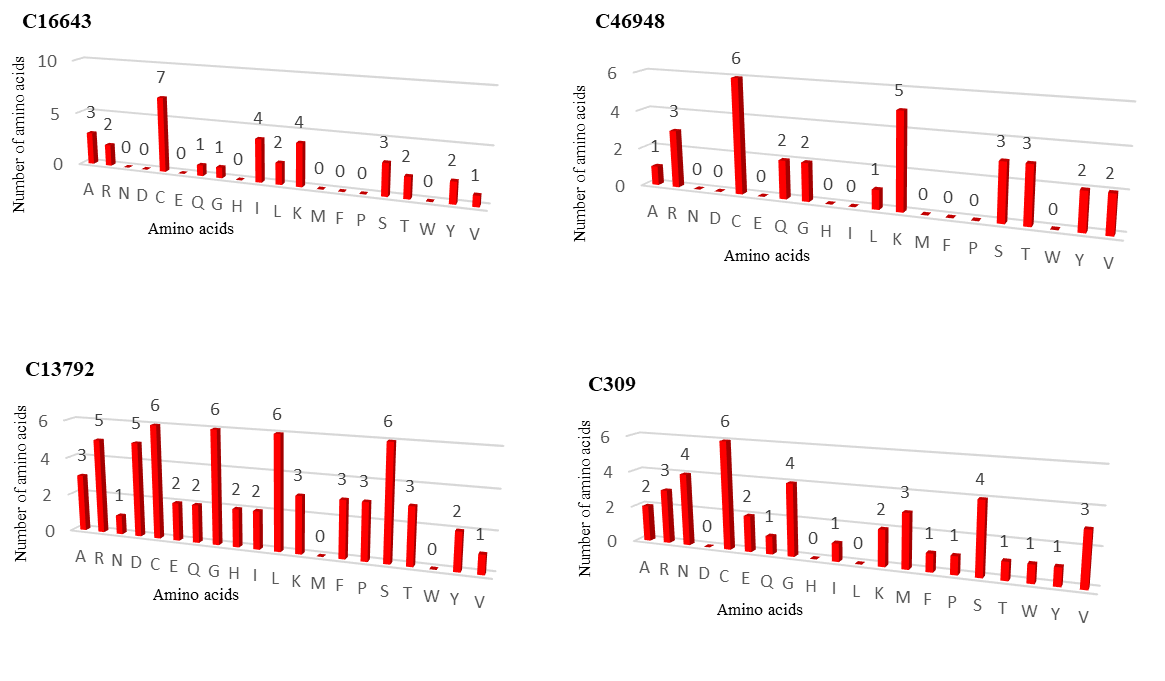
**

**
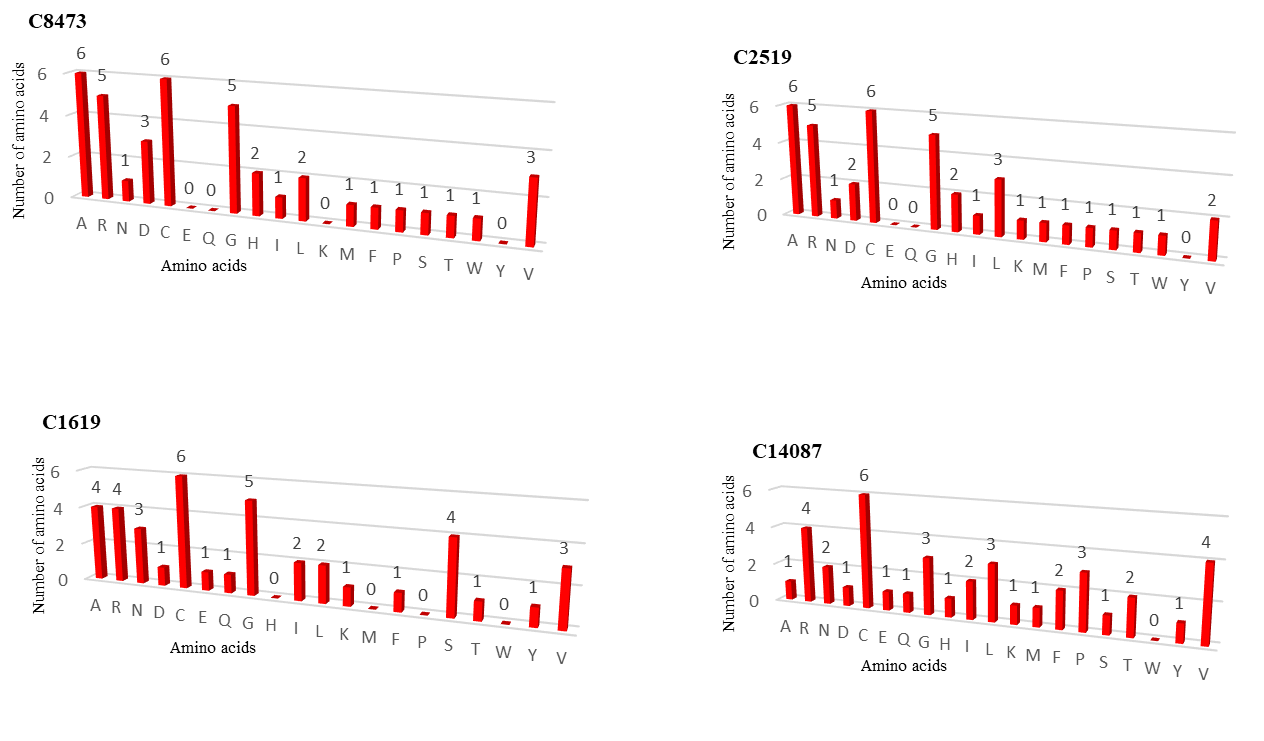

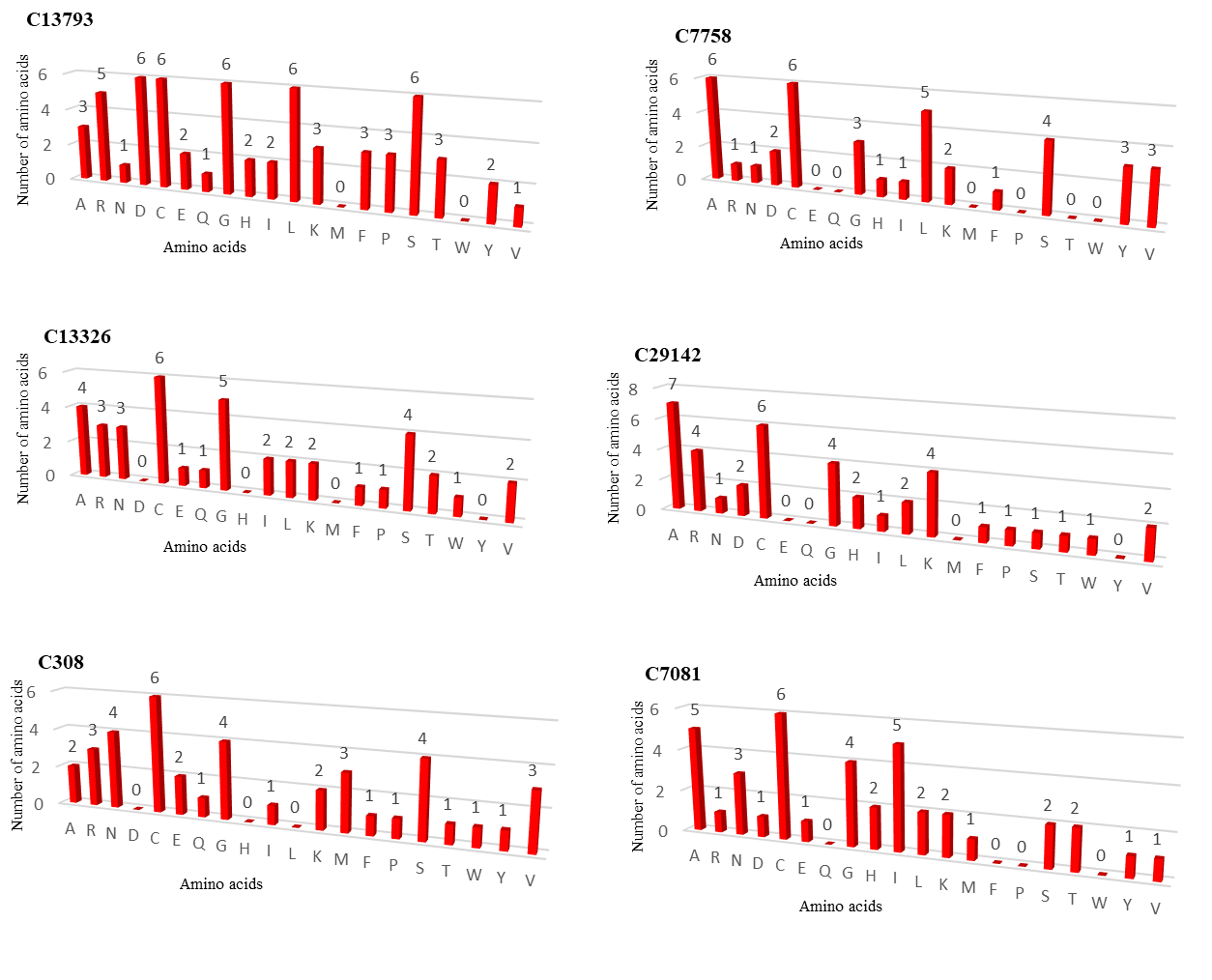

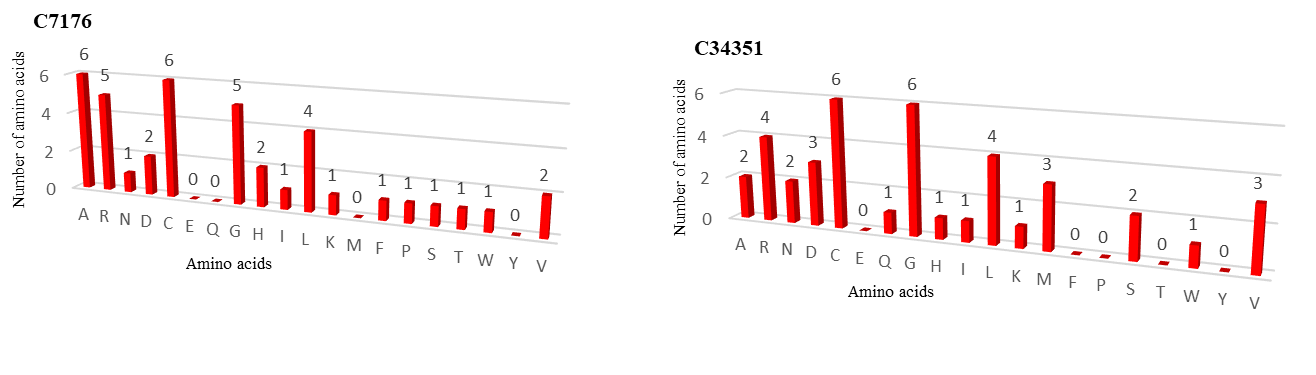

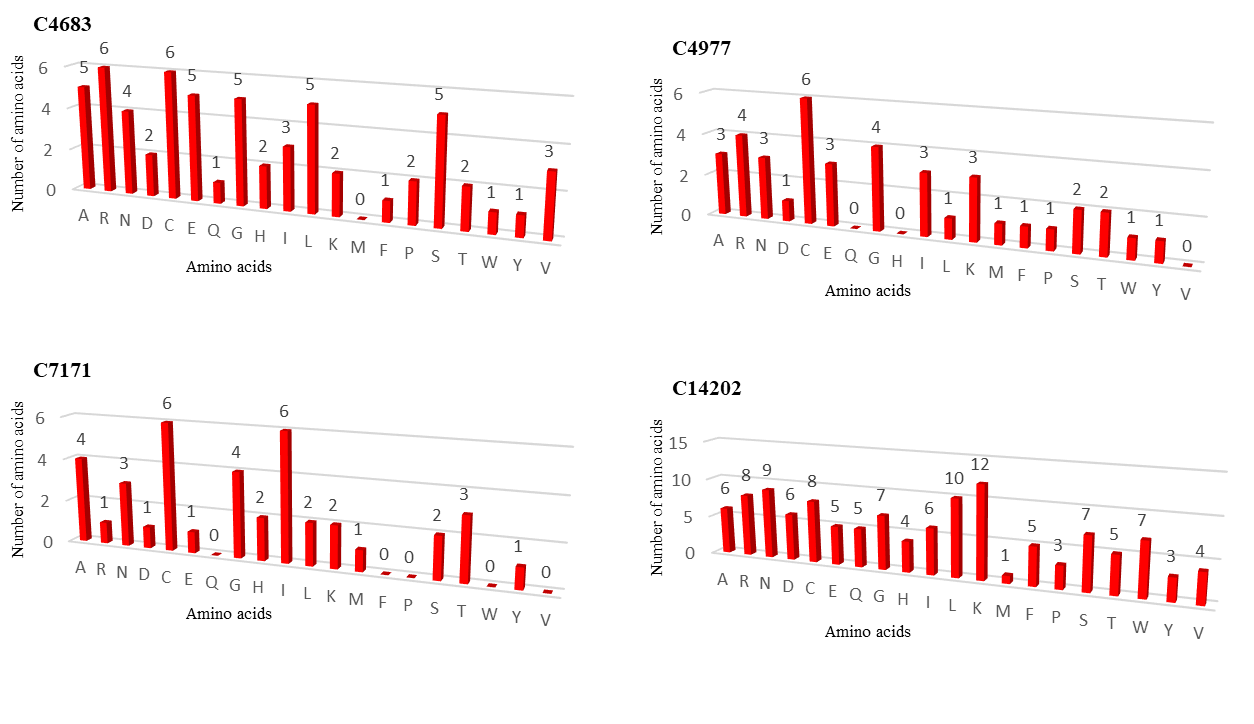

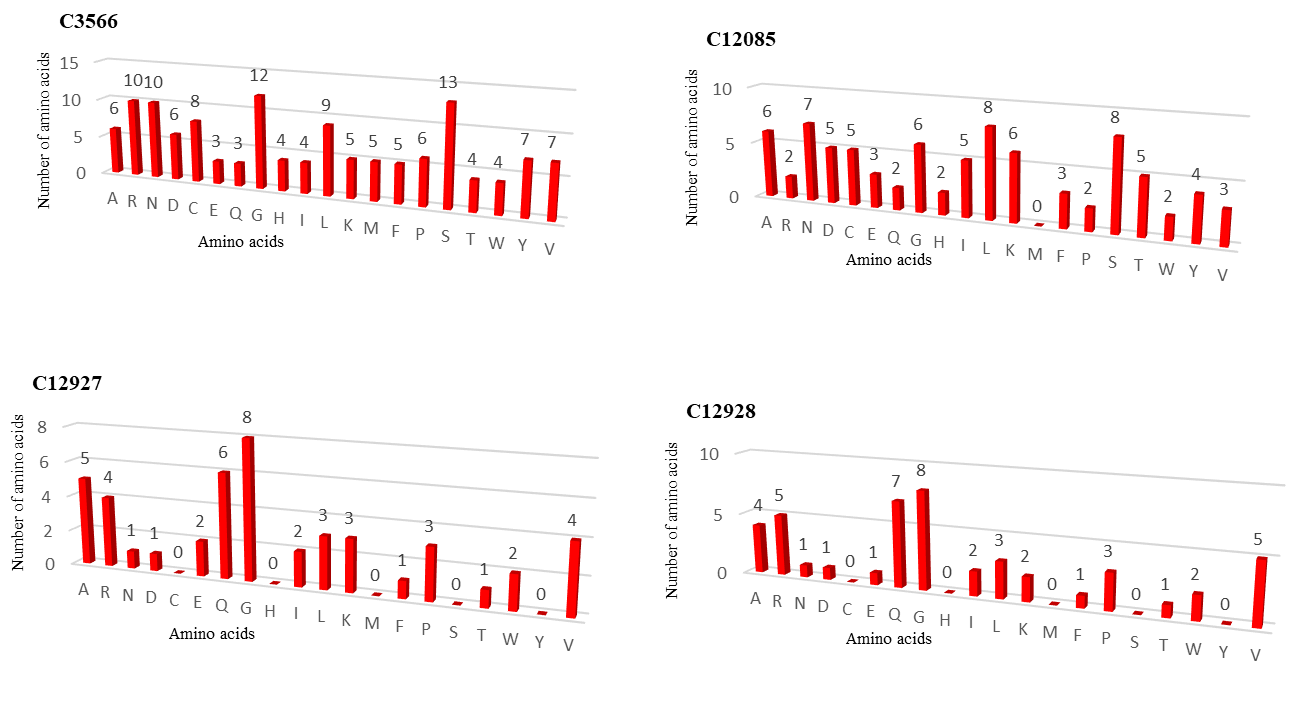

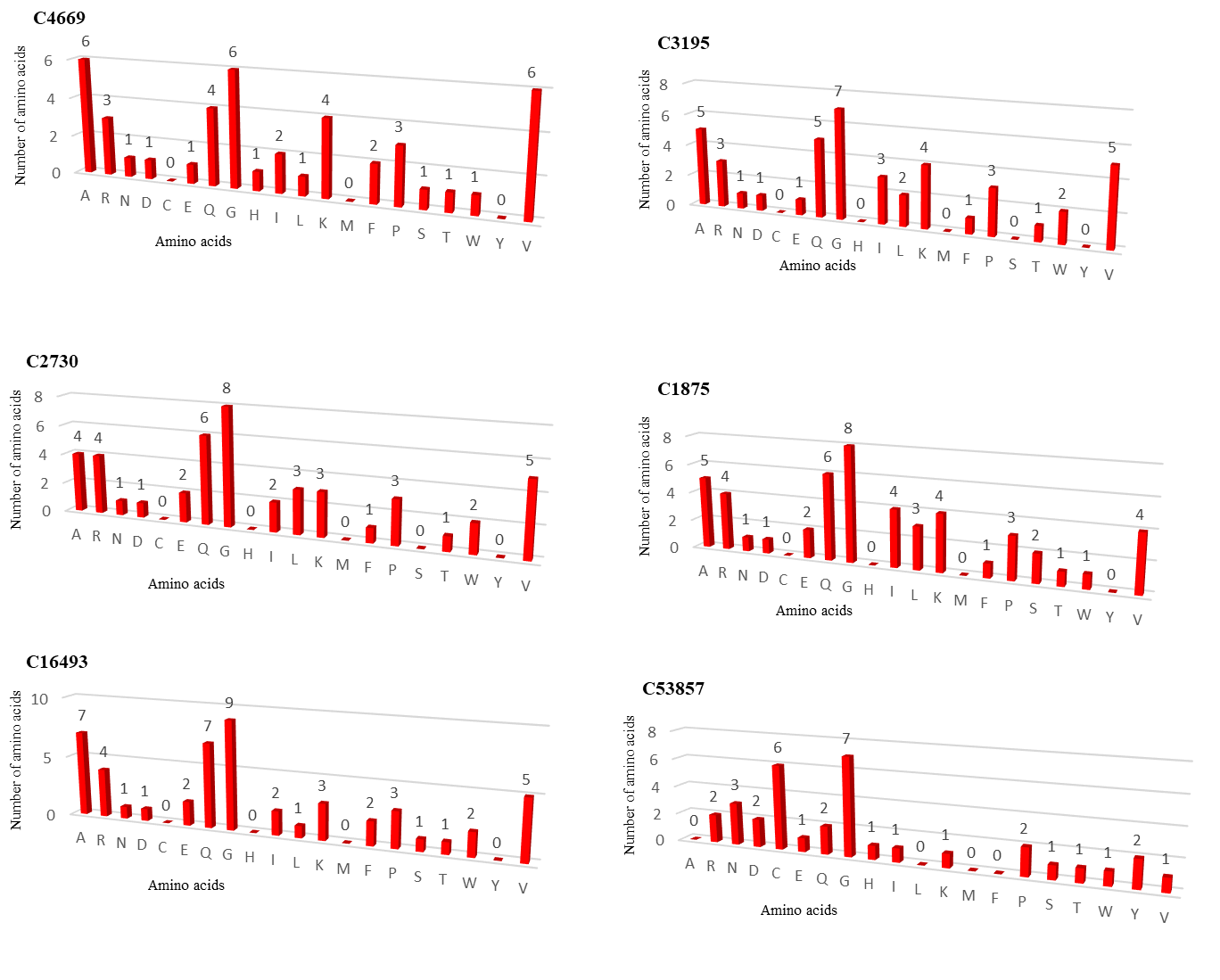

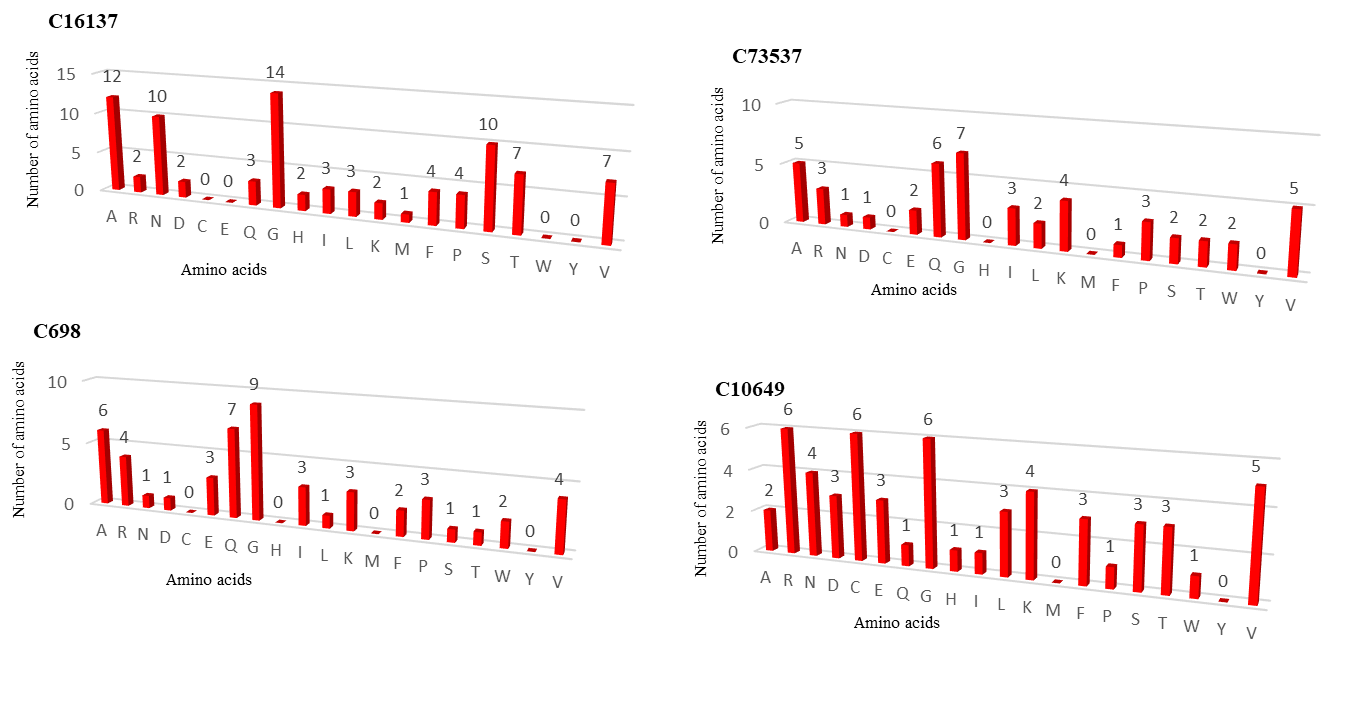

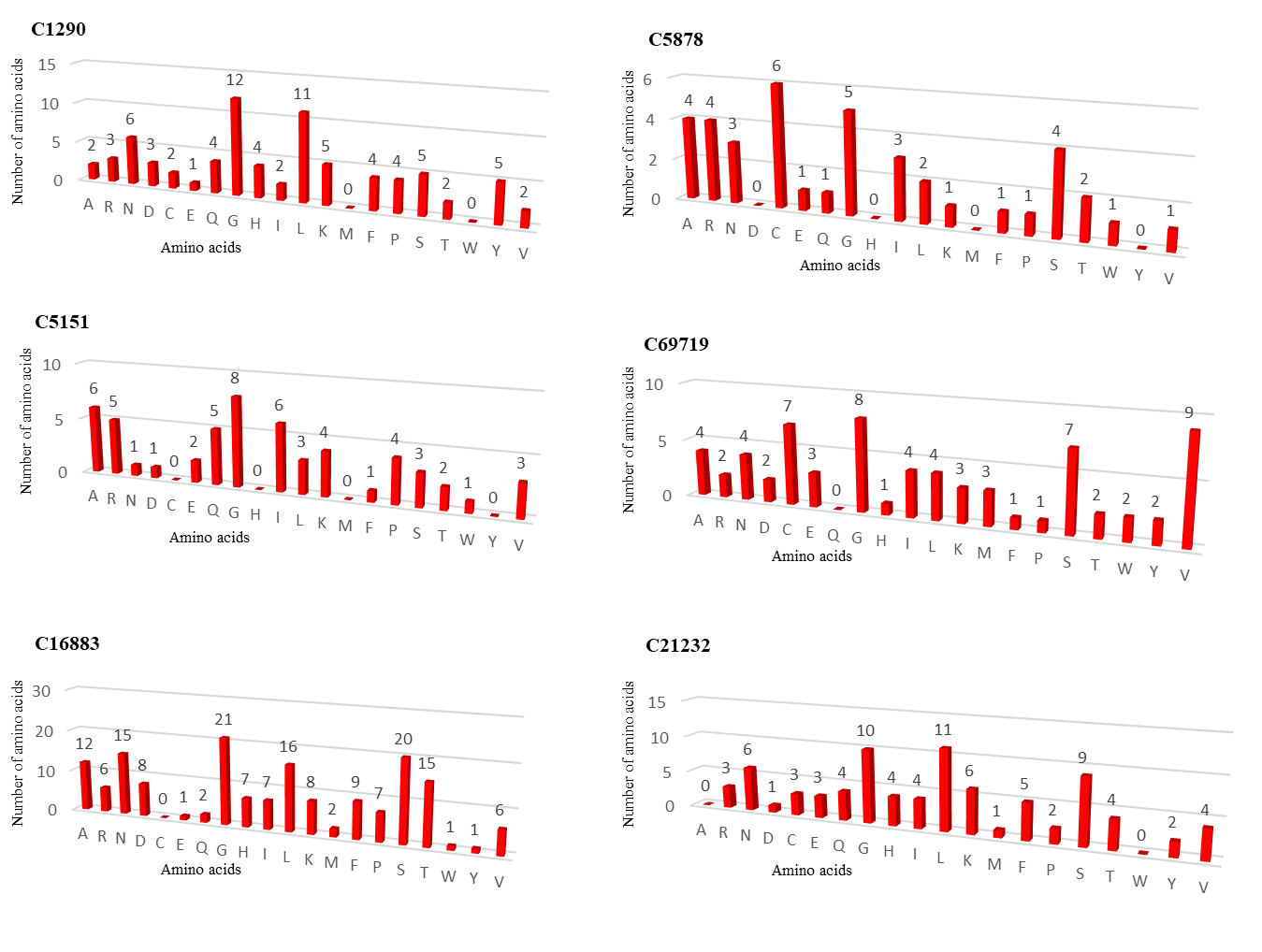

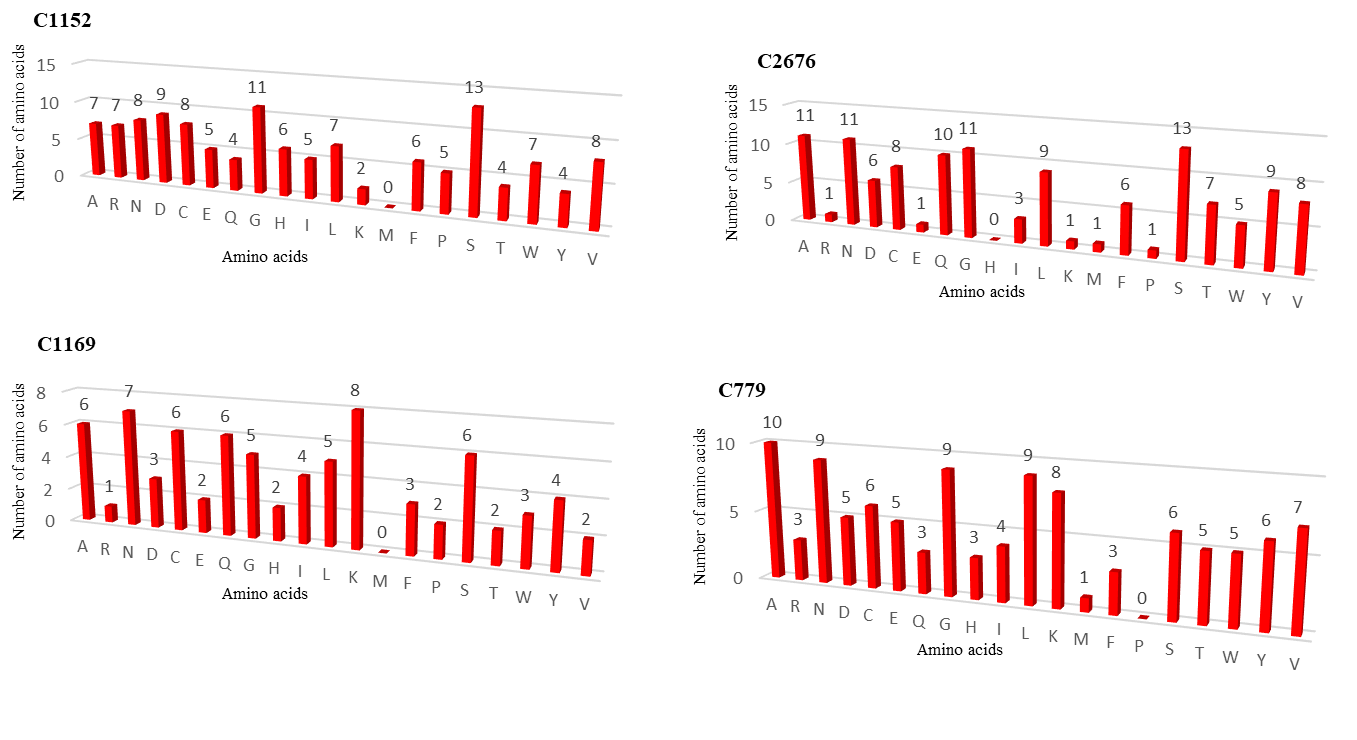

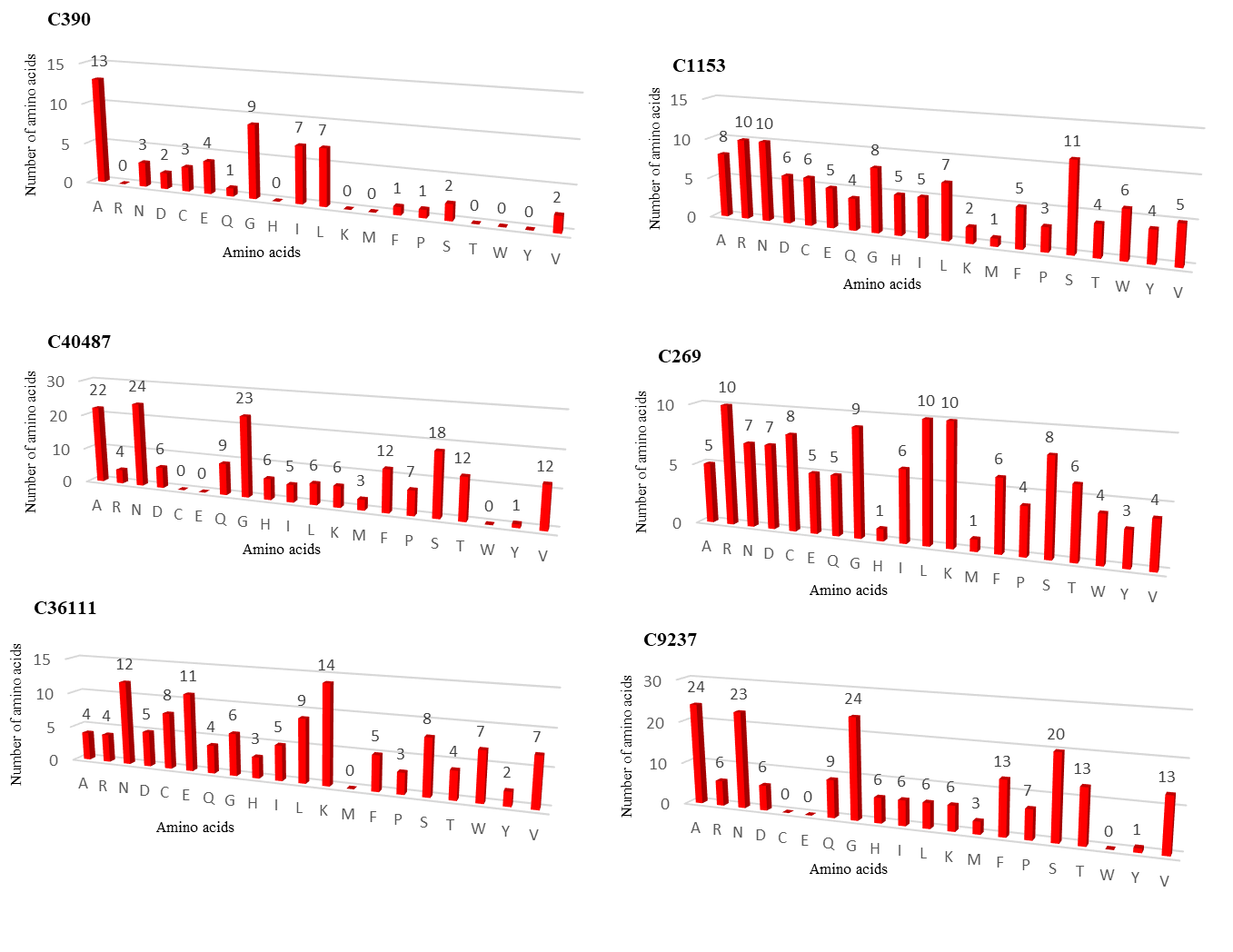

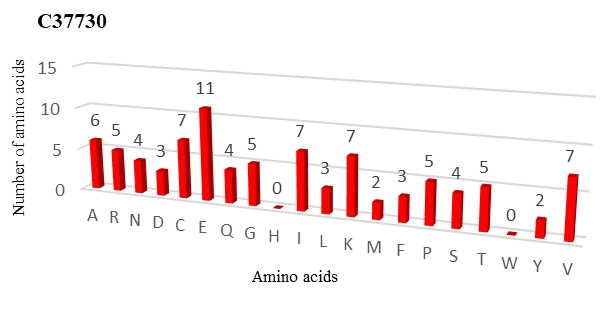
**

**Supplementary Figure 1. Graphical representation of the 57 identified peptides’ amino acid composition.** Bar represents the number of each amino acid for each peptide.


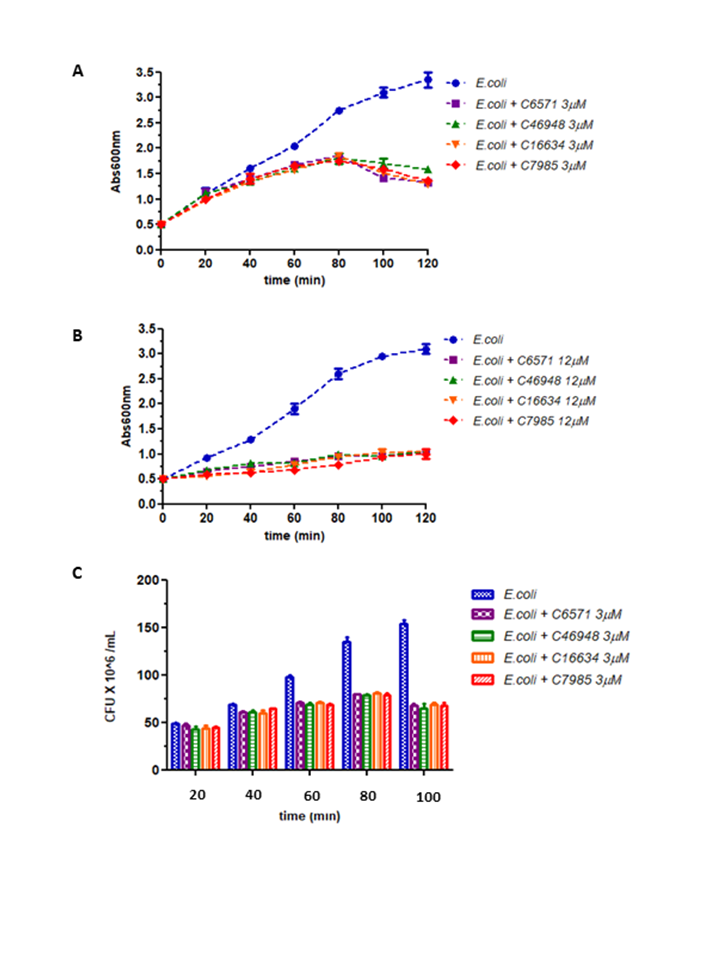


**Supplementary Figure 2.** Bacterial growth profiles under treatment with two different concentrations, 3 μM (A) and 12 μM (B) of each antimicrobial peptide. (C) Cell viability after treatment with 3 μM of each antimicrobial peptide.
